# Comparison of gefitinib plus chemotherapy versus gefitinib alone for advanced non‑small‑cell lung cancer: A meta analysis

**DOI:** 10.1016/j.clinsp.2022.100152

**Published:** 2023-01-19

**Authors:** Min Yi, Ting He, Kaijin Wang, Yonggang Wei

**Affiliations:** a Department of Respiratory and Critical Care Medicine, The People's Hospital of Kaizhou District, China; b Department of Respiratory and Critical Care Medicine, The People's Hospital of Bishan District, China

**Keywords:** NSCLC, Gefitinib, Chemotherapy, Objective response rate, Disease control rate, Progression-free survival, Overall survival, Complication

## Abstract

This study aimed to perform a meta‑analysis comparing the efficacy and safety of gefitinib in combination with chemotherapy versus gefitinib alone in patients with advanced Non‑Small Cell Lung Cancer (NSCLC). We searched databases for clinical studies that reported the efficacy or safety of gefitinib plus chemotherapy in comparison with gefitinib alone. Raw data from included studies were extracted and pooled to calculate the Odds Ratio (OR) for Objective Response Rate (ORR) and Disease Control Rate (DCR), the Hazard Ratio (HR) for Progression-Free Survival (PFS) and Overall Survival (OS), and OR for complication ≥ Grade 3. A total of 10 studies containing 1,528 patients with NSCLC were identified and included in the analysis. Gefitinib plus chemotherapy showed significantly better efficacy in improving ORR (OR = 1.54; 95% CI [Confidence Interval], 1.13‒2.1; p = 0.006), DCR (OR = 1.62; 95% CI 1.14‒2.29; p = 0.007), PFS (HR=1.67; 95% CI 1.45‒1.94; *p* < 0.001) and OS (HR = 1.49; 95% CI 1.2‒1.87; *p* < 0.001) as compared with gefitinib alone. Consistent results were observed in the sub-population with positive EGFR mutation. The combination of gefitinib with chemotherapy had a significantly higher risk of complication (≥ Grade 3) with an OR of 3.29 (95% CI 2.57‒4.21; *p* < 0.001). The findings in the present study suggest that the combination of gefitinib with chemotherapy can provide better disease response and survival outcomes for patients with advanced NSCLC.

## Highlights

• This meta-analysis shows that gefitinib plus chemotherapy showed significantly better efficacy in improving objective response rate, disease control rate, progression-free survival, and OS as compared with gefitinib alone.
• Similar efficacy of gefitinib plus chemotherapy is found in the sub-population with positive EGFR mutation.
• The toxicity of gefitinib plus chemotherapy is also increased but within clinical management.

## Introduction

Lung cancer is the most commonly diagnosed neoplasm, with 2.207 million new cases and 1.79 million deaths worldwide based on global cancer statistics in 2020.^1^^,^^2^ Lung cancer has the highest incidence and mortality in China, with 816,000 new cases and 715,000 associated deaths which accounts for 23.8% of all cancer deaths.^3^ The costs of diagnosis and treatment of lung cancer has huge burden on the shoulders of national health system. Non‑Small Cell Lung Cancer (NSCLC) is the most frequently diagnosed histological subtype which accounts for approximately 85% of all lung cancers.^4^ Up to 40% to 50% patients with NSCLC are diagnosed at advanced and inoperative stages since the symptoms of NSCLC at the early stage are difficult to detect, thus they have to only receive palliative therapy.^5^ The standard initial therapy for these patients with advanced disease was mainly based on combined chemotherapeutic agents such as platinum anticancer drugs. However, the median Overall Survival (OS) following chemotherapy was usually 8‒10 months in most cases. Therefore, improving the treatment strategy for advanced NSCLC is essential.

Individualized therapy for NSCLC has emerged as the key player targeting oncogenic driver mutations.^6^ Globally, the development of NSCLC in 10%‒30% of patients is associated with the gene mutations of Epidermal Growth Factor Receptor (EGFR).^7^ The Asia-Pacific NSCLC population has the highest EGFR mutation frequency of up to approximately 40%.^7^^,^^8^ Particularly, up to 75.3% of lung adenocarcinomas from never smokers in China harbor EGFR mutations.^9^ Currently there have been three generation of EGFR Tyrosine-Kinase Inhibitors (TKIs) approved for the treatment of patients with EGFR-mutant NSCLC: the first-generation reversible EGFR TKIs including gefitinib and erlotinib, the second-generation irreversible EGFR TKIs such as dacomitinib and afatinib, and the recently approved third-generation EGFR TKI, Osimertinib.^10^, ^11^, ^12^ EGFR TKIs can bind to the ATP-binding site of the intracellular tyrosine kinase and suppress the autophosphorylation of EGFR, thus inhibiting the EGFR signaling and tumor progression. In several phase III studies, EGFR TKIs have shown favorable clinical efficacy as compared with platinum-based doublet chemotherapy as first‑line therapy in patients with advanced NSCLC with EGFR mutations, with improved Progression-Free Survival (PFS), response rate, quality of life and acceptable toxicity.^13^^,^^14^

Currently, EGFR-TKIs are the standard initial choice for patients with NSCLC with positive EGFR gene mutation, and gefitinib monotherapy is widely used in East Asia. Although more than half of patients with EGFR-mutant NSCLC initially show response to gefitinib, most of them have to face the fate of acquired drug resistance which is mainly due to the emergence of the T790 M mutation,^15^ resulting in a median PFS of merely 12–14 months. Approximately 30% of the patients might lose the opportunity of subsequent therapy due to the rapid cancer progression. Therefore, to counteract drug resistance and improve prognosis, clinicians have investigated the combination of gefitinib with chemotherapy as a potential breakthrough of the bottleneck of single gefitinib therapy.

Before the last decade, Chen et al. performed two phase II randomized trials investigating the combined therapy of gefitinib with chemotherapeutic agents (vinorelbine and tegafur/uracil, respectively).^16^^,^^17^ Both studies had found that the addition of chemotherapy could produce significantly better Progression-Free Survival (PFS) as compared with gefitinib monotherapy. Since then, there have been emerging studies exploring more combined chemotherapeutic therapies with gefitinib. The NEJ009 study is a recent phase III clinical trial investigating the efficacy of gefitinib alone versus gefitinib plus pemetrexed and carboplatin as the first-line therapy of EGFR mutation-positive patients with advanced NSCLC,^18^ and has received enormous attention since it was reported at the 2018 American Society of Clinical Oncology (ASCO). This randomized trial showed that the combination therapy of gefitinib plus chemotherapy (carboplatin + pemetrexed) had significantly improved Objective Response Rate (ORR) (84% vs. 67%), PFS (20.9 vs. 11.9 months) and OS (50.9 vs. 38.8 months) as compared with gefitinib alone in 345 patients with newly diagnosed metastatic NSCLC with EGFR mutations.^18^ Despite these successes, there has been controversy regarding the efficacy and safety of combination therapy. Yang et al. found that PFS was not significantly different between pemetrexed + cisplatin + gefitinib maintenance therapy versus gefitinib monotherapy in East Asian patients with locally advanced or metastatic NSCLC (p = 0.217).^19^ Besides, they also observed a significantly lower ORR of the combination therapy during the induction period (23.7% vs. 40.7%, p = 0.008).^19^ On the other hand, most of the comparative trials have reported higher incidence of complications in the combination group, especially the complications over grade III.^20^, ^21^, ^22^ Therefore, the efficacy and safety profiles of gefitinib plus chemotherapy versus gefitinib alone in patients with NSCLC need to be further elucidated.

In the present meta‑analysis, we aimed to obtain a more comprehensive understanding of the combined use of gefitinib with chemotherapeutic agents versus gefitinib alone regarding the efficacy and safety in patients with advanced NSCLC.

## Methods

### Literature search

The literature focusing on the combination of gefitinib and chemotherapy in patients with NSCLC in the following databases were searched: PubMed, Embase, Web of Science and Cochrane. The following key words were used for literature search:

For gefitinib: gefitinib, ZD1839 and IRESSA;

For combination of chemotherapy: chemotherapy, chemotherapeutic, combination, combined, plus, intercalating, intercalated, pemetrexed, platinum, carboplatin and paclitaxel;

For NSCLC: NSCLC, lung and pulmonary.

Additional literature search was supplemented by examining the reference list of the literatures identified, especially recent reviews. Endnotes (version X7) was used to manage the literatures. The protocol of this meta-analysis has been registered in the International Prospective Register of Systematic Reviews (PROSPERO, registration ID: CRD42022302886). Two authors independently evaluated the eligibility of literatures for inclusion, and the dissonance of the result was dissolved via discussion with the third author. The human-based studies were considered suitable for inclusion with the following criteria: 1) Comparative study investigating gefitinib versus gefitinib plus chemotherapy; 2) Patients with advanced NSCLC; 3) The outcome was Objective Response Rate (ORR), Disease Control Rate (DCR), Progression-Free Survival (PFS) or Overall Survival (OS). Exclusion criteria: 1) Duplicate literatures; 2) Case report or case series; 3) With less than 20 patients; 4) Not in English; 5) Pre-print without peer-review.

### Data extraction

Two authors independently collected the raw data. As for the mismatch of raw data collected by the two authors, a third author would preside over a discussion until consensus was obtained. The following data were collected: first author, time of publication, study location, size and age of population, rate of EGFR mutation, participant selection, combined chemotherapy drug, type of tumor, stage of cancer, previous treatment, study design, follow up time, number of ORR and DCR, HR (Hazard Ratio) and 95% CI of HR for PFS and OS, number of complications with Grade ≥3. If HR for PFS and OS was not provided in the article, time-to-event data were extracted from Kaplan-Meier curve by using the software Engauge, and HR was then calculated *via* the method provided by Tierney et al.^23^

### Definitions

The response rate was calculated according to Response Evaluation Criteria in Solid Tumors (RECIST), version 1.1. ORR was defined as the rate of Complete Response (CR) + Partial Response (PR), while DCR was defined as the rate of CR + PR + Stable Disease (SD).

To incorporate the HR of all included studies with subtle differences of definitions of PFS and OS, the following definitions were used in this meta-analysis: PFS was defined as the time from study randomization or treatment start or the baseline radiological assessment until disease progression (objective or subjective deterioration) or death as a result of any cause, whichever came first. Patients who had not progressed or died were censored on the time of their last cancer assessment. OS was defined as period from the date of random assignment or treatment start to the date of death as a result of any cause. Patients who had not died or lost follow up were censored on the time of their last follow up.

Adverse events were scored according to the National Cancer Institute Common Terminology Criteria for Adverse Events (CTCAE, version 4.03).

### Quality assessment

Newcastle-Ottawa Scale (NOS) is a quality assessment tool for observational studies that has been suggested by the Cochrane Collaboration. For included studies, NOS was used to assess the quality. The results were visualized by presenting each score of 1 as green circle, 0 as red circle and unavailable score as yellow circle. The studies were considered as high quality if they scored > 6-points, moderate quality if they scored 5 or 6 points, and poor quality if they scored < 5-points.

### Data synthesis and statistical analysis

To guarantee the reliability of the results, analysis was performed by two authors independently. Dissonance was resolved by discussion. To compare the efficacy and safety of gefitinib plus chemotherapy versus gefitinib alone, the *metan* module of the STATA software, version 15 (Stata Corporation, College Station, TX) was used to calculate the pooled Odds Ratio (OR) for ORR, DCR and complication, and HR for PFS and OS following the random effects model. The *Z* test was used to determine the significance of OR and HR, and *p* < 0.05 was considered statistically significant. The data were presented as pooled estimate with 95% CI and plotted as forest plot. The *I*^2^ statistic and p-value were calculated to assess the heterogeneity.

### Sensitivity analysis

To evaluate the influence of every study on the pooled estimates, sensitivity analysis was performed by omitting one study at each analysis using the *metaninf* module of the STATA software. Results were presented as forest plot to show the influence of each study omitted.

### Publication bias analysis

Publication bias was estimated by using *Egger's* test and funnel plot via the *metabias* module and *metafunnel* module of the STATA software, respectively. The p-value of *Egger's* test < 0.05 was considered as significant publication bias. The asymmetry of funnel plot was also helpful to evaluate the possibility of underreported result.

### Subgroup analysis

Subgroup analysis was performed by using the *metan* module of the STATA software, according to the type of tumor, stage of cancer, previous treatment, special type of population, study design, total number of patients, average age and follow up duration. Besides, for PFS and OS, subgroup analysis was also conducted based on whether the raw data (HR) were extracted from the Kaplan-Meier curve.

### Additional analysis for the sub-population with positive EGFR mutation

As an EGFR TKI, gefitinib was initially used to treat patients with positive EGFR mutation. However, not all the included patients in this meta-analysis had confirmed the status of EGFR mutation. To further compare the efficacy and safety of gefitinib plus chemotherapy versus gefitinib alone in patients with positive EGFR mutation, we also extracted the data of the sub-population with positive EGFR mutation if these were reported. OR for ORR and DCR, HR for PFS and OS were calculated based on the additionally extracted data.

## Results

### Search results and study characteristics

**Fig. 1:**
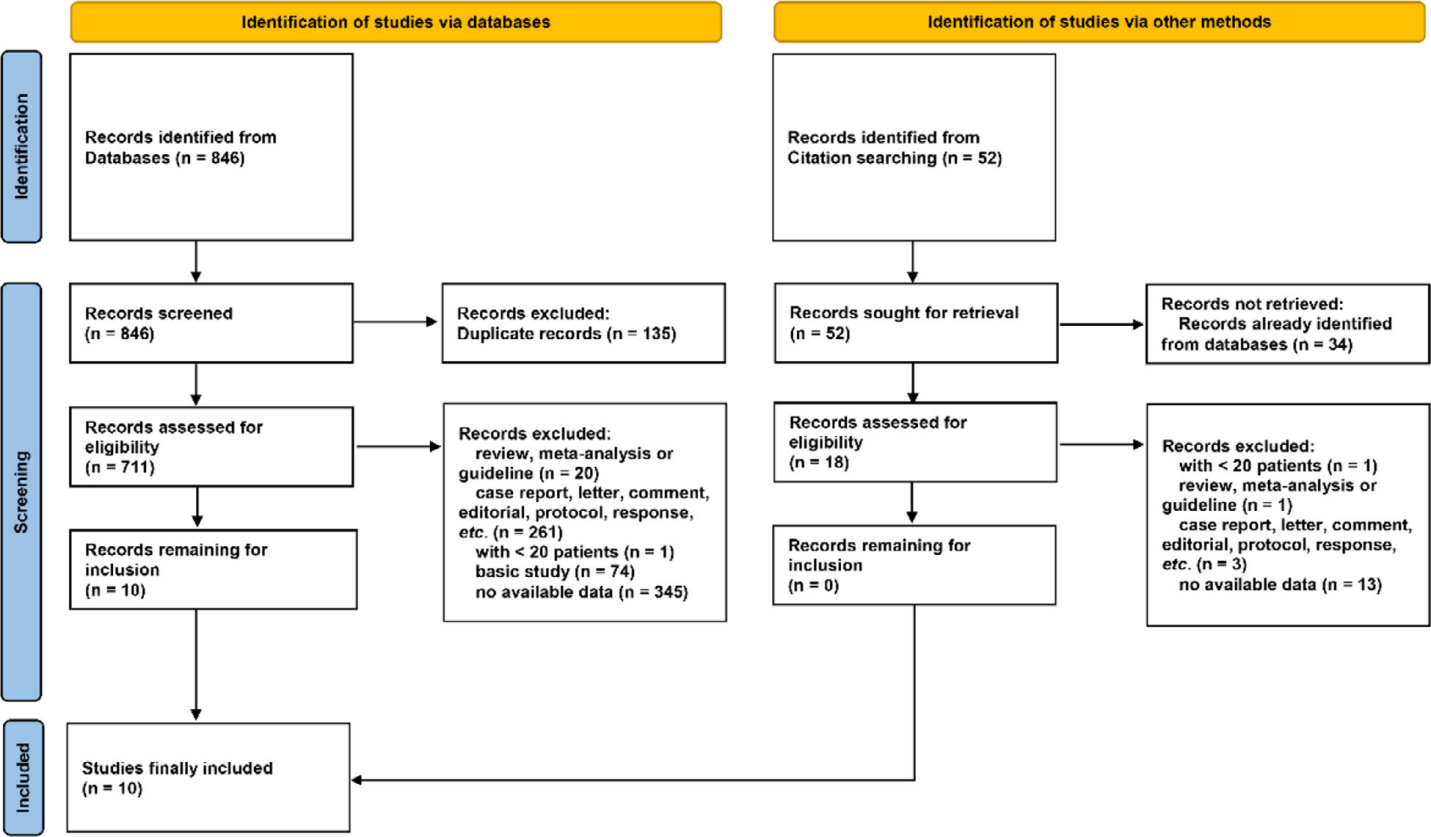
**Flowchart of study selection.** Records were identified via databases (*n* = 846) and other methods (citation searching, *n* = 52). After screening, a total of 10 and 0 records were considered eligible for inclusion from databases and other methods, respectively. Finally, 10 studies were included in the present meta-analysis.

As shown in Fig. 1, a total of 846 literatures were initially identified *via* databases searching, of which 135 duplicates were excluded. After screening by reviewing title, abstract and full-text, 10 literatures were considered eligible. Besides, 52 literatures were identified by citation searching, but none was suitable for inclusion. Thus a total of 10 literatures^16^, ^17^, ^18^, ^19^, ^20^, ^21^, ^22^^,^^24^, ^25^, ^26^ were eventually included in this meta-analysis. Notably, the two studies by Yang et al. in 2014 and 2015 reported PFS and OS of the same patient population, respectively. Thus, the two studies were incorporated and presented as one study (“2014&2015 Yang”) in the following analysis.

The characteristics of included literatures are listed in Table 1. A total of 1,528 patients were included. All studies were performed in Asia, mostly in China. Three studies included not only patients with positive EGFR mutations but also those without. The type of tumor was adenocarcinoma, nonsquamous NSCLC and NSCLC in 3, 2 and 4 studies, respectively. The study^19^^,^^24^ by Yang et al. included only nonsmoker or light former smoker. Only one study was retrospectively performed, others were prospective and randomized studies. The combined chemotherapeutic drugs included vinorelbine, tegafur, uracil, pemetrexed, cisplatin, carboplatin. The combination of gefitinib + pemetrexed + platinumcontaining drugs were the most frequently used therapy in 4 of the 9 studies. The patients in 5 studies were chemonaive or without other prior systemic anticancer therapy for advanced disease. Two studies included patients previously treated with chemotherapy. The previous treatment of patients in the other 2 studies were unknown.

**Table 1:** Characteristics of included studies.

Study ID	Study location	Rate of EGFR mutation	Type of tumor	Stage of cancer	Special type of population	Prospective and randomized	Combined treatment	Number of patients	Previous treatment
2007 Chen^16^	China	50%	Lung adenocarcinoma	IV	None	Yes	Vinorelbine	48	previous chemotherapy with >= 2 regimens
2011 Chen^17^	China	67%	Lung adenocarcinoma	IIIB/IV	None	Yes	Tegafur/Uracil	115	failed previous chemotherapy
2014 and 2015 Yang^19^^,^^24^^a^	Asian multicentre	68%	NSCLC	IIIB/IV	Nonsmoker/Light former smoker	Yes	Pemetrexed +cisplatin	236	chemonaive
2016 An^20^	China	100%	NSCLC	IIIB/IV	None	Yes	Pemetrexed	90	N/A
2016 Cheng^21^	Asian multicentre	100%	Nonsquamous NSCLC	IV/Recurrent	None	Yes	Pemetrexed	191	no prior systemic chemotherapy, immunotherapy, or biologic therapy
2017 Han^22^	China	100%	Lung adenocarcinoma	IIIB/IV	None	Yes	Pemetrexed +Carboplatin	81	no prior systemic anticancer therapy for advanced disease
2019 Zhang^25^	China	100%	NSCLC	III/IV	None	No	Cisplatin	92	no prior surgery, chemotherapy, radiotherapy, or immunotherapy
2019 Noronha^26^	India	100%	NSCLC	IIIB/IV	None	Yes	Pemetrexed +Carboplatin	334	N/A
2020 Hosomi^18^	Japan	100%	Nonsquamous NSCLC	IIIB/IV/Recurrent	None	Yes	Pemetrexed +Carboplatin	341	no prior chemotherapy

a The two studies by Yang et al. in 2014 and 2015 reported progression-free survival and overall survival of the same patient population, respectively. Thus, the two studies were considered as one in the present analysis. EGFR, Epidermal Growth Factor Receptor; NSCLC, Non-Small Cell Lung Cancer; N/A, Not Available.

### Quality assessment

**Fig. 2:**
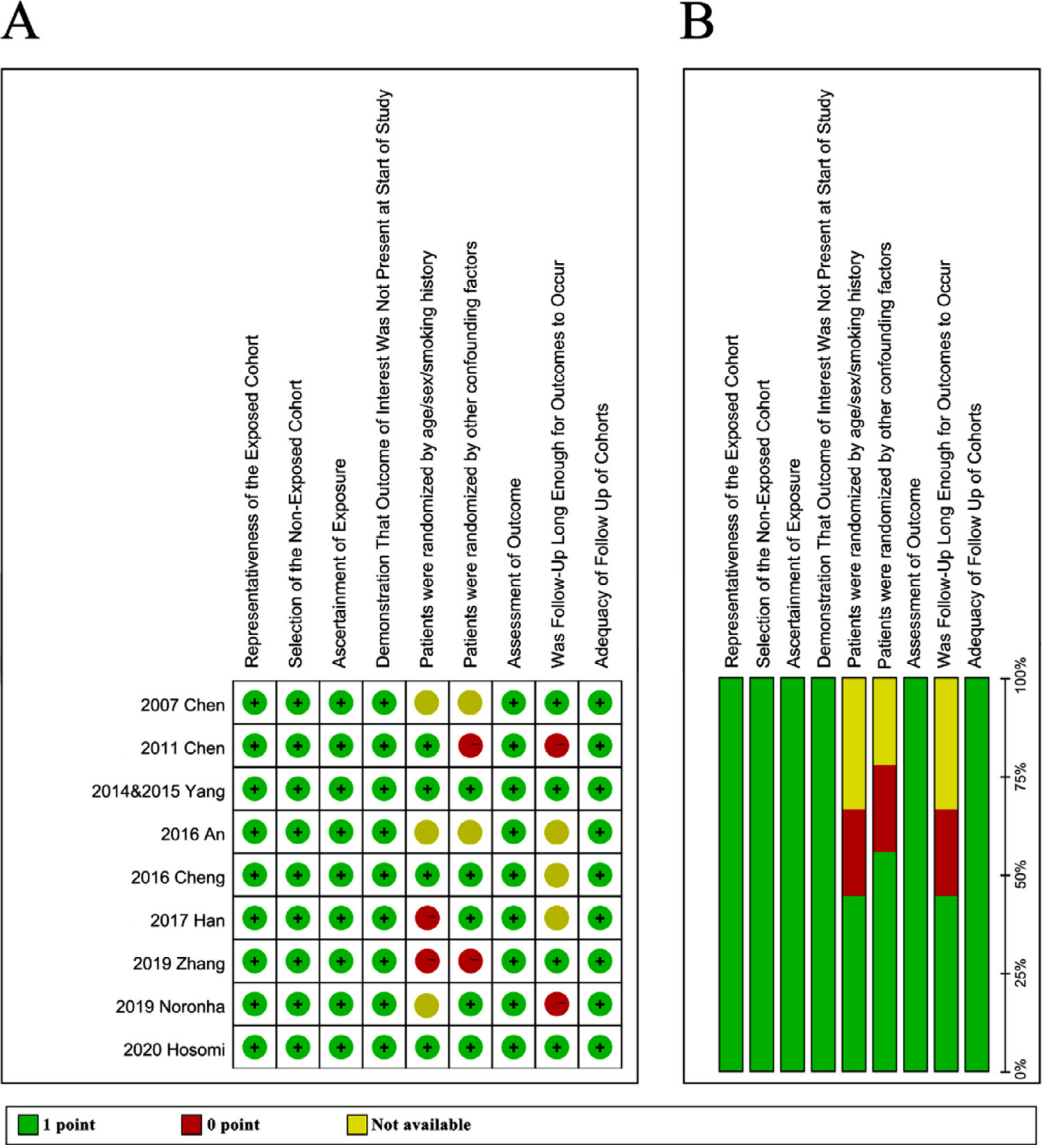
**Quality assessment of included studies.** The quality of included studies was assessed by the Newcastle Ottawa scale (NOS). The following 9 aspects were evaluated: 1) Representativeness of the exposed cohort; 2) Selection of the non-exposed cohort; 3) Ascertainment of exposure; 4) Demonstration that outcome of interest was not present at start of study; 5) Patients were randomized by age/sex/smoking history; 6) Patients were randomized by other confounding factors; 7) Assessment of outcome; 8) Was follow-up long enough for outcomes to occur; 9) Adequacy of follow-up of cohorts. (A) The figure shows the authors' judgements about each aspect of quality item for each included study. Note: the two studies by Yang et al. in 2014 and 2015 reported progression-free survival and overall survival of the same patient sample, respectively. Thus, the two studies were considered as one, and presented as ‘2014&2015 Yang’ in the present analysis. (B) The results are also presented as percentages across all included studies.

As depicted in Fig. 2A and B, most of the studies (8 out of 9) were considered as high quality with NOS score > 6. The study by An et al. was considered as moderate quality with NOS score of 6 due to unavailable detail of patient randomization and follow up duration. Notably, these factors were also the most frequent reason for 0 score in these studies.

### ORR

#### Main finding

**Fig. 3:**
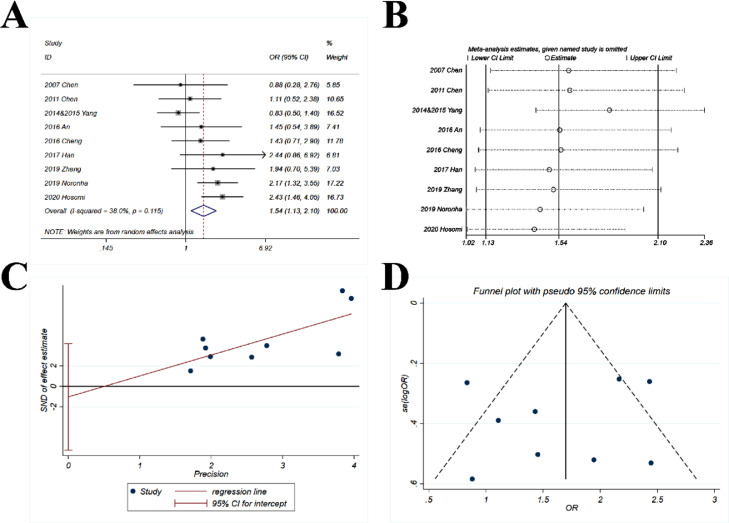
**Comparison of ORR (objective response rate) of gefitinib in combination with chemotherapy versus gefitinib alone.** (A) The forest plot shows the OR (Odds Ratio) of ORR of gefitinib in combination with chemotherapy versus gefitinib alone. OR > 1 indicates gefitinib in combination with chemotherapy has higher probability of ORR as compared with gefitinib alone. (B) Sensitivity analysis was performed by omitting one study at each analysis. The result of each analysis is also presented as the forest plot. (C) The Egger's regression test and (D) Funnel plot were used to detect publication bias.

The pooled OR (1.54; 95% CI 1.13‒2.1; p = 0.006) suggested that the combination of chemotherapy and gefitinib significantly increased the probability of objective response rate for 1.54-fold as compared with gefitinib alone (Fig. 3A). The heterogeneity was minor and non-significant, with *I^2^* = 38% and p = 0.115.

#### Sensitivity analysis

The sensitivity analysis showed that the ORs ranged from 1.4 to 1.82 (Fig. 3B), and the study by Yang et al. had more impact on the pooled OR. However, the results were basically stable, with all ORs > 1.

#### Publication bias

The p-value for *Egger's* test was 0.652 (Fig. 3C) and the funnel plot (Fig. 3D) showed a good symmetry, indicating that a significant publication bias was highly unlikely.

#### Subgroup analysis

As shown in Supplementary Fig. S1 to S9, the subgroup of type of tumor (NSCLC), stage of cancer (IIIB/IV), previous treatment (no chemotherapy), total number of patients (≥ 100), average age (< 60) and follow up time (≥ 20-months) had enhanced heterogeneity with *I^2^* > 50%, suggesting that these factors were potential source of heterogeneity. However, since the overall heterogeneity was minor (*I^2^* = 38%), the impacts of these factors were considered non-significant. Besides, the ORs in all subgroups with more than 2 studies were all > 1, further supporting the main finding.

### DCR

#### Main finding

**Fig. 4:**
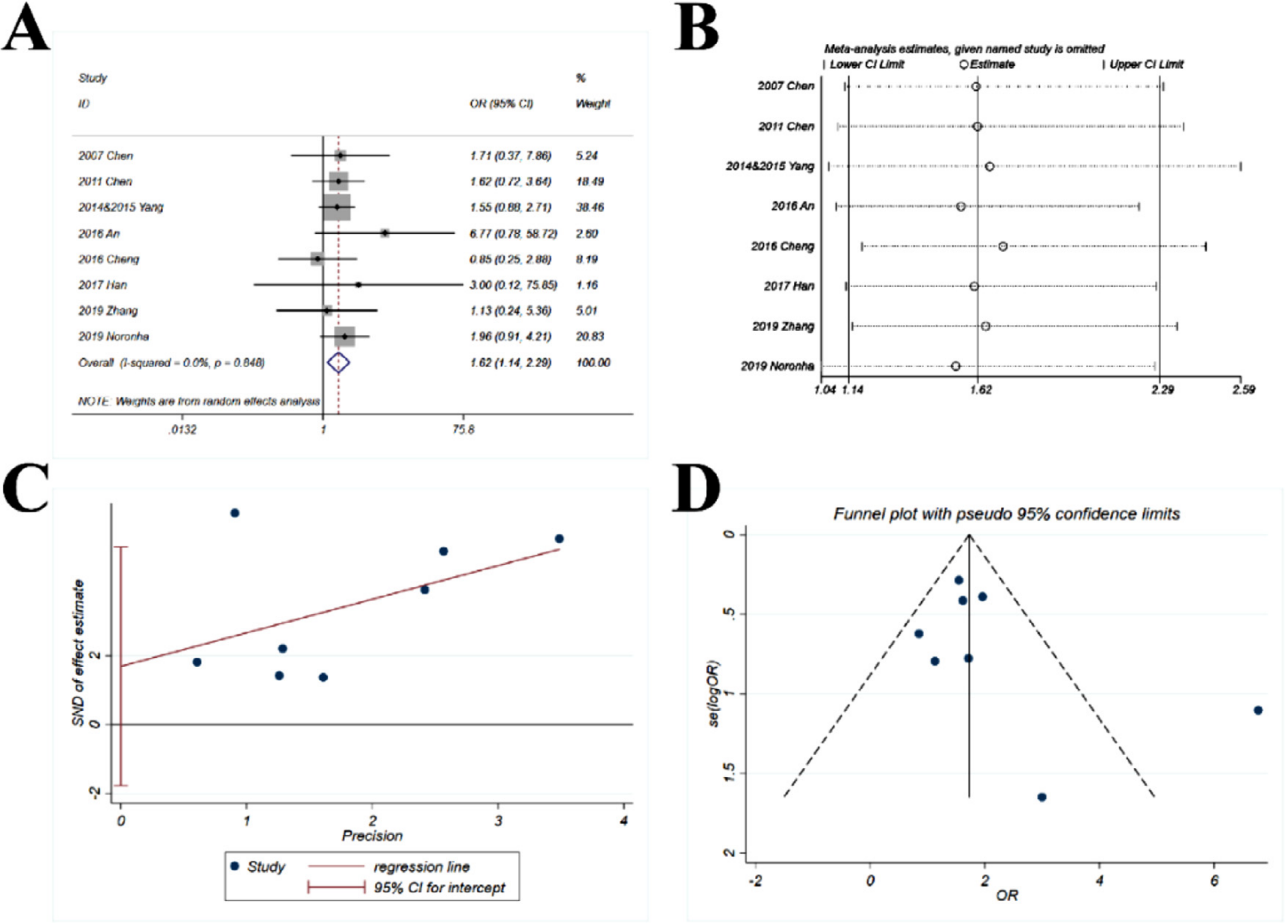
**Comparison of DCR (disease control rate) of gefitinib in combination with chemotherapy versus gefitinib alone.** (A) The forest plot shows the OR (Odds Ratio) of DCR of gefitinib in combination with chemotherapy versus gefitinib alone. OR > 1 indicates gefitinib in combination with chemotherapy has higher probability of DCR as compared with gefitinib alone. (B) Sensitivity analysis was performed by omitting one study at each analysis. The result of each analysis is also presented as the forest plot. (C) The Egger's regression test and (D) Funnel plot were used to detect publication bias.

The data of DCR were provided in 8 studies. The pooled OR (1.62; 95% CI 1.14‒2.29; p = 0.007) suggested that the combination of chemotherapy and gefitinib significantly increased the probability of disease control rate for 1.62-fold as compared with gefitinib alone (Fig. 4A). No heterogeneity was observed with *I^2^* = 0% and p = 0.848.

#### Sensitivity analysis

The sensitivity analysis showed that the ORs ranged from 1.54 to 1.71 (Fig. 4B), which were quite stable with only minor variations as compared with the pooled OR (1.62).

#### Publication bias

The p-value for *Egger's* test was 0.276 (Fig. 4C) and the funnel plot (Fig. 4D) was slightly asymmetric, with only one study away from the main part of the funnel.

#### Subgroup analysis

As shown in Supplementary Fig. S10 to S18, there was no moderate (*I^2^* > 50%) or substantial (*I^2^* > 75%) heterogeneity in all subgroups, further demonstrating the homogeneity of the 8 studies in terms of DCR. The ORs in all subgroups with more than 2 studies were all > 1.

### PFS

#### Main finding

**Fig. 5:**
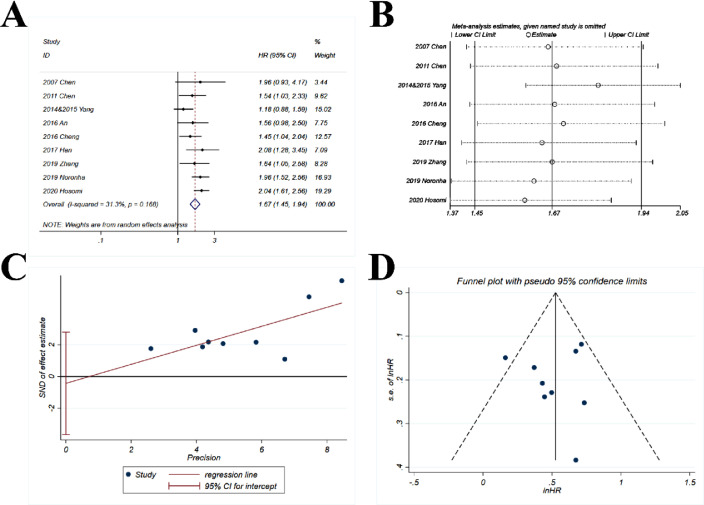
**Comparison of PFS (progression-free survival) of gefitinib in combination with chemotherapy versus gefitinib alone.** (A) The forest plot shows the HR (Hazard Ratio) of PFS of gefitinib in combination with chemotherapy versus gefitinib alone. HR > 1 indicates gefitinib in combination with chemotherapy has higher probability of progression-free survival as compared with gefitinib alone. (B) Sensitivity analysis was performed by omitting one study at each analysis. The result of each analysis is also presented as the forest plot. (C) The Egger's regression test and (D) Funnel plot were used to detect publication bias.

The pooled HR (1.67; 95% CI 1.45‒1.94; *p* < 0.001) indicated that the combination of chemotherapy and gefitinib significantly improved the possibility of survival without disease progression for 1.67-fold as compared with gefitinib alone (Fig. 5A). Minor and non-significant heterogeneity was observed with *I^2^* = 31.1% and p = 0.168.

#### Sensitivity analysis

The sensitivity analysis showed that the HRs ranged from 1.59 to 1.81 (Fig. 5B), which were quite stable with only minor variations as compared with the pooled HR (1.67).

#### Publication bias

The p-value for *Egger's* test was 0.761 (Fig. 5C) and the funnel plot (Fig. 5D) was basically symmetric.

#### Subgroup analysis

As shown in Supplementary Fig. S19 to S28, the subgroup of type of tumor (NSCLC and nonsquamous NSCLC), stage of cancer (IIIB/IV), previous treatment (no chemotherapy), total number of patients (≥ 100), average age (< 60) and follow up time (≥ 20-months) had moderate heterogeneity with *I^2^* > 50%, suggesting that these factors were potential source of heterogeneity. However, since the overall heterogeneity was minor (*I^2^* = 31.1%), the impacts of these factors were considered non-significant. Besides, the HRs for PFS were both > 1 with *p* < 0.05 in the subgroups of HRs either obtained from the raw data or calculated from the Kaplan-Meier curve.

### OS

#### Main finding

**Fig. 6:**
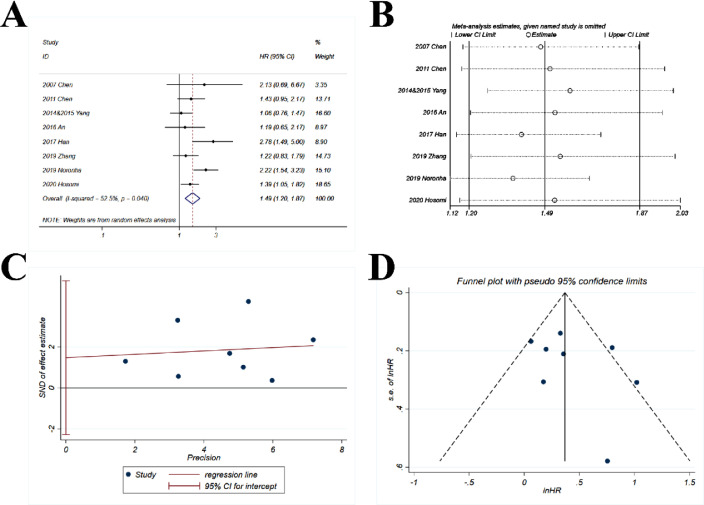
**Comparison of OS (overall survival) of gefitinib in combination with chemotherapy versus gefitinib alone.** (A) The forest plot shows the HR (Hazard Ratio) of OS for gefitinib in combination with chemotherapy versus gefitinib alone. HR > 1 indicates gefitinib in combination with chemotherapy has higher probability of overall survival as compared with gefitinib alone. (B) Sensitivity analysis was performed by omitting one study at each analysis. The result of each analysis is also presented as the forest plot. (C) The Egger's regression test and (D) Funnel plot were used to detect publication bias.

The pooled HR (1.49; 95% CI 1.2‒1.87; *p* < 0.001) indicated that the combination of chemotherapy and gefitinib significantly improved the possibility of overall survival for 1.49-fold as compared with gefitinib alone (Fig. 6A). Significant moderate heterogeneity was observed with *I^2^* = 52.5% and p = 0.04.

#### Sensitivity analysis

The sensitivity analysis showed that the HRs ranged from 1.37 to 1.59 (Fig. 6B) with only minor variations as compared with the pooled HR (1.49).

#### Publication bias

The p-value for *Egger's* test was 0.372 (Fig. 6C) and the funnel plot (Fig. 6D) was basically symmetric.

#### Subgroup analysis

As shown in Supplementary Fig. S29 to S38, the subgroup of type of tumor (NSCLC), stage of cancer (IIIB/IV), previous treatment (unavailable and no treatment), study design (prospective & randomized), total number of patients (< 100 and ≥ 100), average age (< 60), follow up time (unavailable) and the source of OS (obtained from raw data) showed heterogeneity with *I^2^* > 50%, suggesting that these factors were potential source of heterogeneity. Besides, the HRs for OS were both > 1 with *p* < 0.05 in the subgroups of HRs either obtained from the raw data or calculated from the Kaplan-Meier curve.

### Complication

#### Main finding

**Fig. 7:**
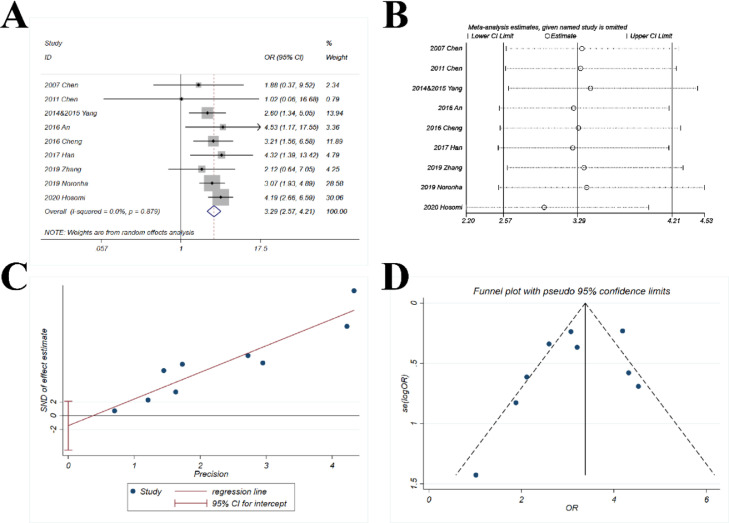
**Comparison of complication ≥ Grade 3 of gefitinib in combination with chemotherapy versus gefitinib alone.** (A) The forest plot shows the OR (Odds Ratio) of complication ≥ Grade 3 of gefitinib in combination with chemotherapy versus gefitinib alone. OR > 1 indicates gefitinib in combination with chemotherapy has higher probability of complication ≥ Grade 3 as compared with gefitinib alone. (B) Sensitivity analysis was performed by omitting one study at each analysis. The result of each analysis is also presented as the forest plot. (C) The Egger's regression test and (D) Funnel plot were used to detect publication bias.

The pooled OR (3.29; 95% CI 2.57‒4.21; *p* < 0.001) suggested that the combination of chemotherapy and gefitinib significantly enhanced the risk of complication ≥ Grade 3 for 3.29-fold as compared with gefitinib alone (Fig. 7A). No heterogeneity was observed with *I^2^* = 0% and p = 0.879.

#### Sensitivity analysis

The sensitivity analysis showed that the ORs ranged from 2.96 to 3.42 (Fig. 7B). The study by Hosomi et al. had greater impact on the pooled OR yet the results were basically stable, with all ORs > 2.9.

#### Publication bias

The p-value for *Egger's* test was 0.368 (Fig. 7C) and the funnel plot (Fig. 7D) was basically symmetric.

### Sub-population with positive EGFR mutation

**Fig. 8:**
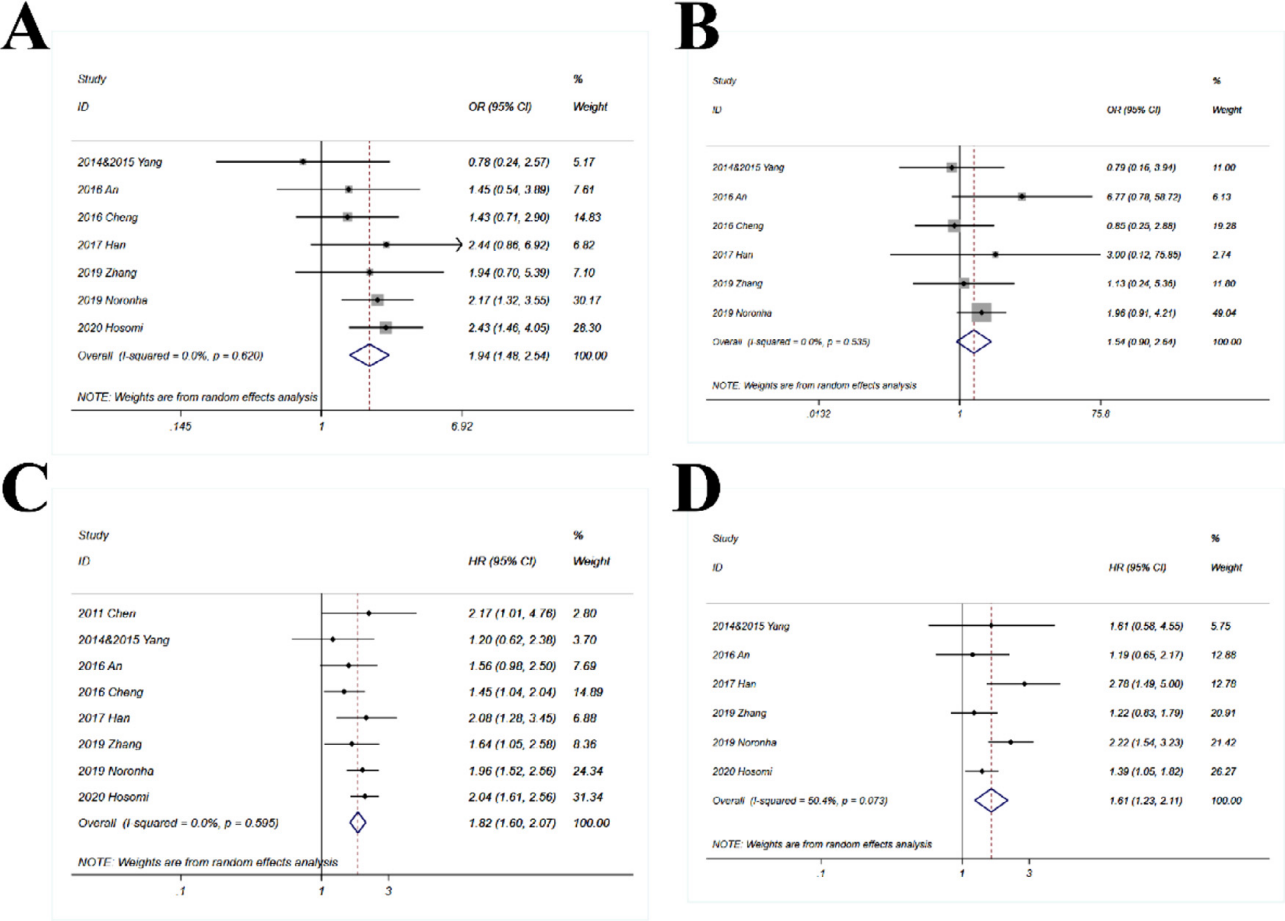
**Comparison of gefitinib in combination with chemotherapy versus gefitinib alone in patients with EGFR mutations.** The following outcomes of gefitinib in combination with chemotherapy and gefitinib alone were compared in patients with EGFR mutations: (A) Objective Response Rate (ORR); OR (Odds Ratio) > 1 indicates gefitinib in combination with chemotherapy has higher probability of ORR as compared with gefitinib alone; (B) Disease Control Rate (DCR); OR > 1 indicates gefitinib in combination with chemotherapy has higher probability of DCR as compared with gefitinib alone; (C) Progression-Free Survival (PFS); HR > 1 indicates gefitinib in combination with chemotherapy has higher probability of progression-free survival as compared with gefitinib alone; and (D) Overall Survival (OS); HR > 1 indicates gefitinib in combination with chemotherapy has higher probability of overall survival as compared with gefitinib alone.

Only one study did not provide the data of sub-population with positive EGFR mutation. Thus 8 studies were included in this part. The pooled OR for ORR (1.94; 95% CI 1.48‒2.54; *p* < 0.001) suggested that the combination of chemotherapy and gefitinib significantly enhanced the probability of objective response rate for 1.94-fold as compared with gefitinib alone in the sub-population with positive EGFR mutation (Fig. 8A). The pooled OR for DCR was 1.54 (95% CI 0.9‒2.64) which was close to statistically significant (p = 0.11) (Fig. 8B). The combination of chemotherapy and gefitinib also significantly improved the survival outcomes as compared with gefitinib alone in the sub-population with positive EGFR mutation, with HR = 1.82 for PFS (95% CI 1.6‒2.07; *p* < 0.001; Fig. 8C) and 1.61 for OS (95% CI 1.23‒2.11; *p* < 0.001; Fig. 8D).

## Discussion

In summary, the findings the present meta-analysis suggest that the combination of gefitinib with chemotherapy can improve the disease response and survival outcomes for patients with advanced NSCLC. However, the treatment benefit is accompanied with increased possibility of complication of Grade ≥ 3.

The improved tumor response rate and survival outcomes in the combination group can be attributable to the additional chemotherapy. The sensitivity to EGFR TKIs in patients with advanced NSCLC is closely related with the somatic mutations of EGFR gene. However, EGFR mutated, and non-mutated cancer cells can concurrently exist in a considerable proportion of NSCLC, leading to the heterogeneity of NSCLC cells which results in a reduced response to gefitinib. Quite a percentage of EGFR mutated NSCLC patients can acquire resistance to EGFR TKIs after the first line TKI therapy for approximately one year. The intratumoral genetic heterogeneity of a specific target pathway such as EGFR should be an important concern when treating NSCLC with molecular targeted therapy. Therefore, there have been several studies investigating the effect of chemotherapy on the sensitivity to EGFR TKIs. Silvia et al. reported that in PC9 cells and in PC9 xenografts the combination of gefitinib and pemetrexed prevented gefitinib resistance mediated by the T790M mutation or Epithelial-to-Mesenchymal Transition (EMT) in PC9 and HCC827 cells, respectively, when pemetrexed was the first treatment, given alone or together with gefitinib.^27^ Sequential use of vinorelbine followed by gefitinib was also reported to enhance the antitumor effect in NSCLC cell lines which were poorly responsive to reversible EGFR TKIs.^28^ The expression of activated EGFR and its downstream pathway genes indicated that the enhanced cytotoxic function of the vinorelbine and gefitinib sequential treatment was accompanied by inhibition of EGFR, AKT and ERK1/2. The heterogeneity of NSCLC and the development of TKIs resistance are key factors that hinder response to TKIs and survival, and this constitutes the theoretical rationale for combining chemotherapy with nonoverlapping mechanisms of anti-tumor effects. The combination of gefitinib and chemotherapy might represent a promising first-line option for advanced NSCLC.

The increased risk of complication associated with combination chemotherapy might also be a concern. Most reports have observed the increase in toxicity with combination chemotherapy.^21^ However, these toxicities were mostly clinically manageable, as reflected by the high adherence and relative dose-intensity of both gefitinib and chemotherapeutic drugs. Besides, to avoid hematological and gastrointestinal toxicities, the administration schedule and course of chemotherapy + gefitinib can also be re-considered and adjusted.^22^

There were several strengthens of the present meta-analysis. Firstly, most of the included studies were prospective and randomized trials, which result in the favorable quality assessment. Secondly, the heterogeneity was zero or minor in most of the analysis. No significant publication bias was observed in this analysis. These could also be attributed to the high quality of included studies. Thirdly, we used 4 indices (ORR, DCR, PFS and OS) to thoroughly investigate the efficacy of combination therapy. The results of these 4 indices showed high consistency. All these strengthens had increased the accuracy and reliability of the present findings.

## Conclusion

Based on the results of the present meta-analysis suggests that combination therapy using gefitinib plus chemotherapy could improve the ORR, DCR, PFS and OS relative to gefitinib alone for advanced NSCLC. The toxicity is also increased but within clinical management. The combination of gefitinib plus chemotherapy represents a promising first-line option for advanced NSCLC and should be further investigated in future research.

## Author's contributions

(I) Conception and design: MY and YW. (II) Administrative support: KW. (III) Provision of study materials or patients: MY and TH. (IV) Collection and assembly of data: MY, TH and KW. (V) Data analysis and interpretation: All authors. (VI) Manuscript writing: All authors. (VII) Final approval of manuscript: All authors.

## Ethics approval and consent to participate

Not applicable.

## Consent for publication

Not applicable.

## Availability of data and materials

All data generated or analyzed during this study are included in this published article and its supplementary information files.

## Funding

None.

## Conflicts of interest

The authors declare no conflicts of interest.

## Appendix Supplementary materials

Image, application 1
