# Supplementary material for: Comparison of gefitinib plus chemotherapy versus gefitinib alone for advanced non‑small‑cell lung cancer: A meta analysis
Source: Clinics (Sao Paulo). 2023 Jan 19;78:100152. doi: 10.1016/j.clinsp.2022.100152 (PMC9868856; doi:10.1016/j.clinsp.2022.100152)

CLINICS-D-22-00249_Supplementary Material

**Figure S1** Subgroup analysis for ORR (Objective Response Rate) by study location.


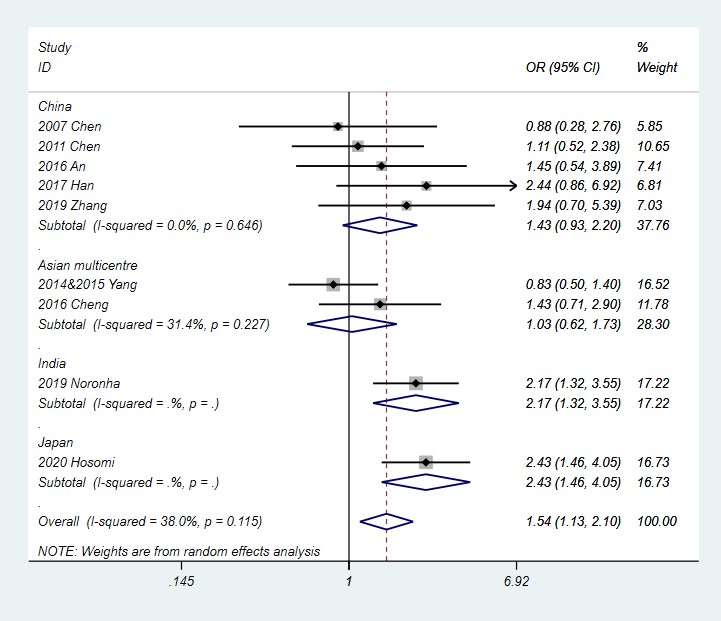


**Figure S2** Subgroup analysis for ORR (Objective Response Rate) by type of tumor.


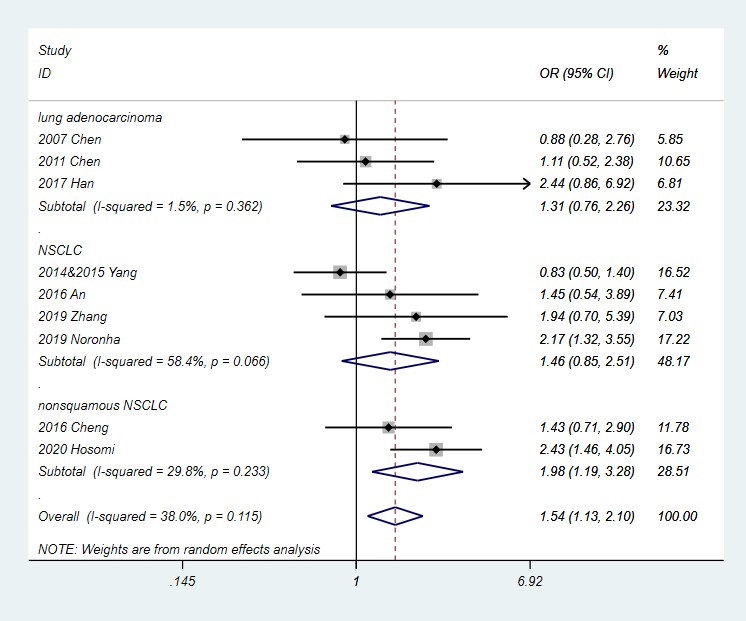


**Figure S3** Subgroup analysis for ORR (Objective Response Rate) by stage of cancer.


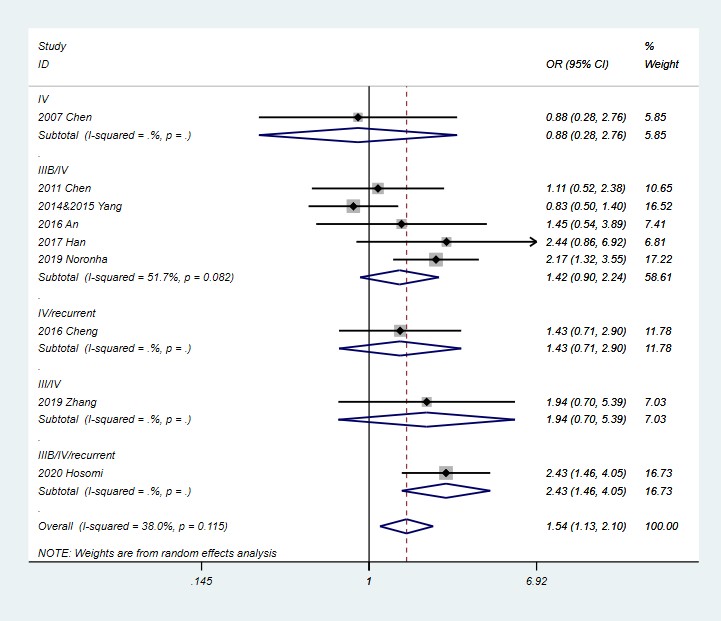


**Figure S4** Subgroup analysis for ORR (Objective Response Rate) by previous treatment.


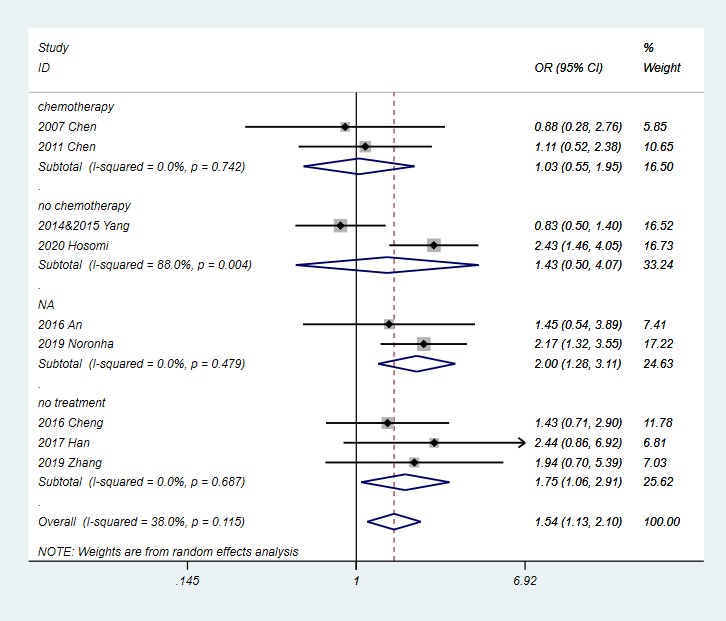


**Figure S5** Subgroup analysis for ORR (Objective Response Rate) by special type of population.


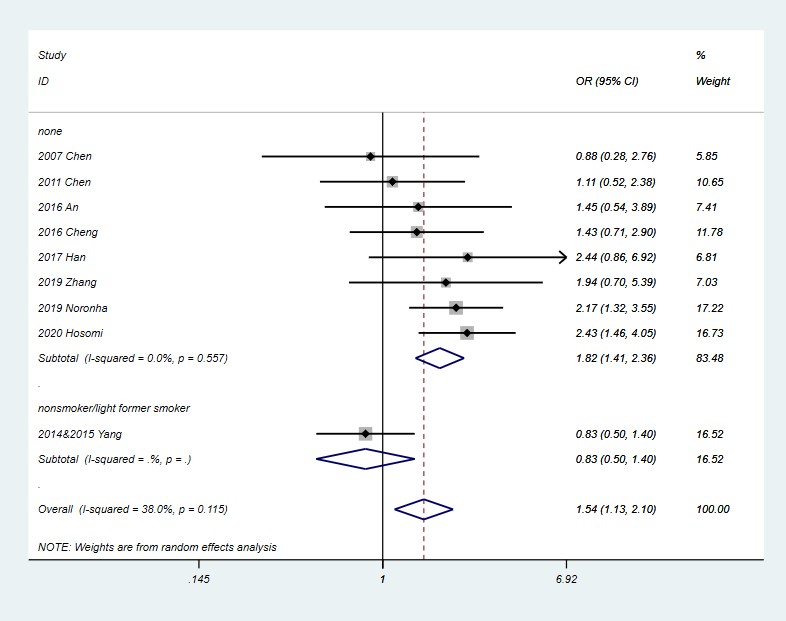


**Figure S6** Subgroup analysis for ORR (Objective Response Rate) by study design.


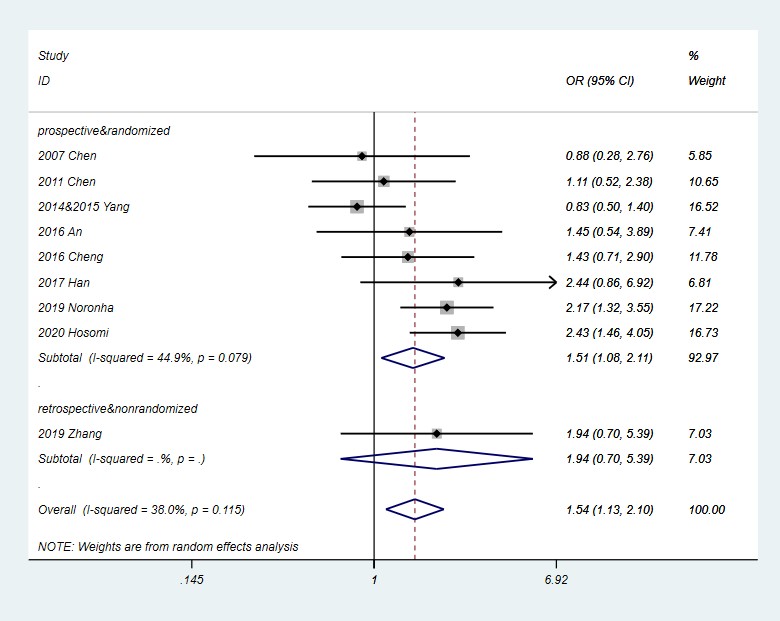


**Figure S7** Subgroup analysis for ORR (Objective Response Rate) by total number of patients.


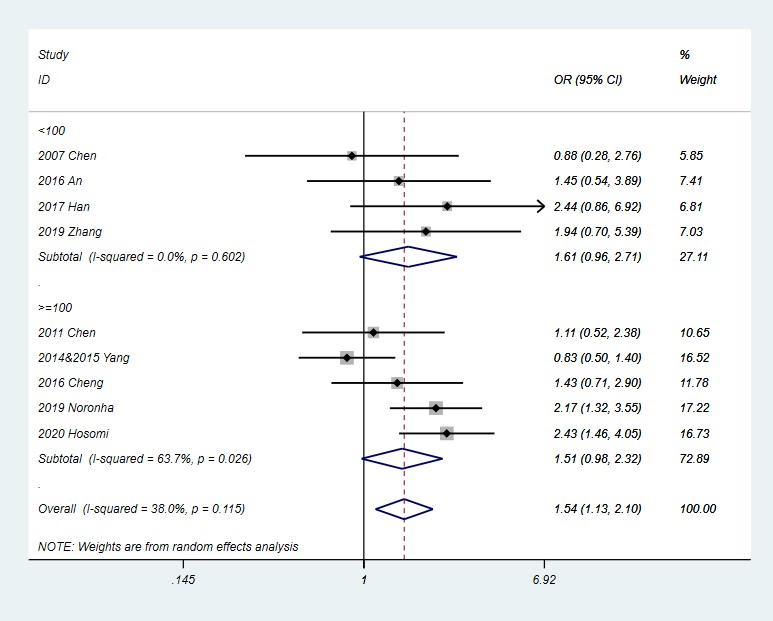


**Figure S8** Subgroup analysis for ORR (Objective Response Rate) by average age.


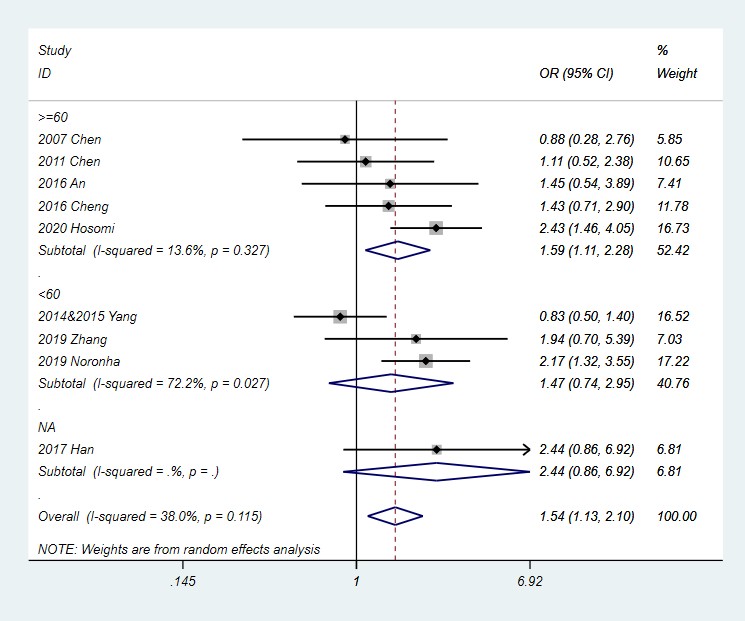


**Figure S9** Subgroup analysis for ORR (Objective Response Rate) by follow up time.


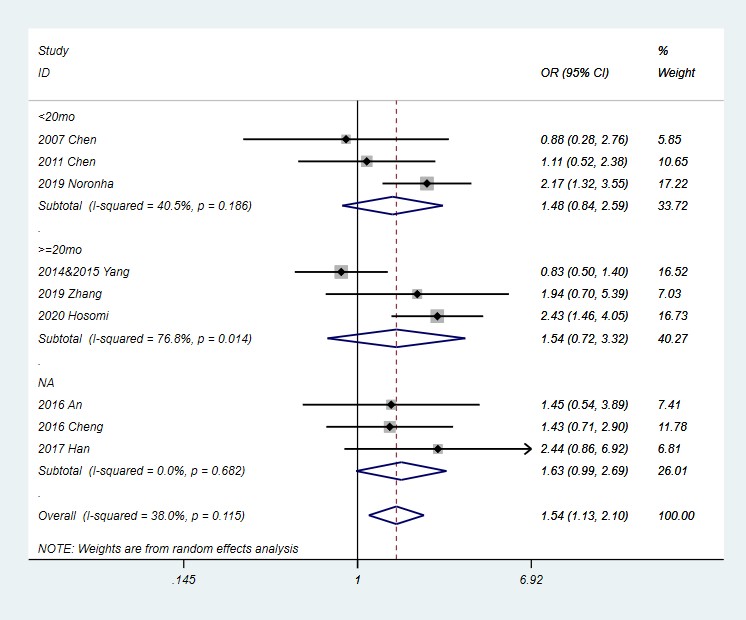


**Figure S10** Subgroup analysis for DCR (Disease Control Rate) by study location.


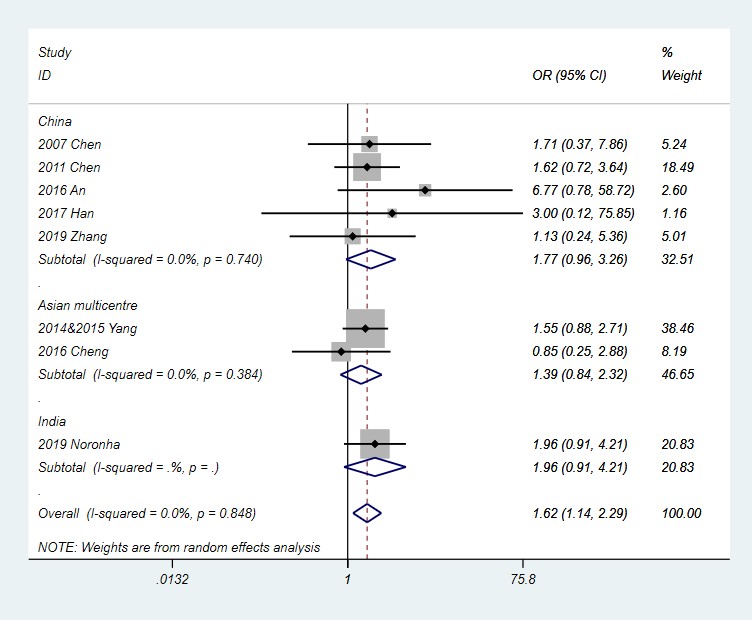


**Figure S11** Subgroup analysis for DCR (Disease Control Rate) by type of tumor.


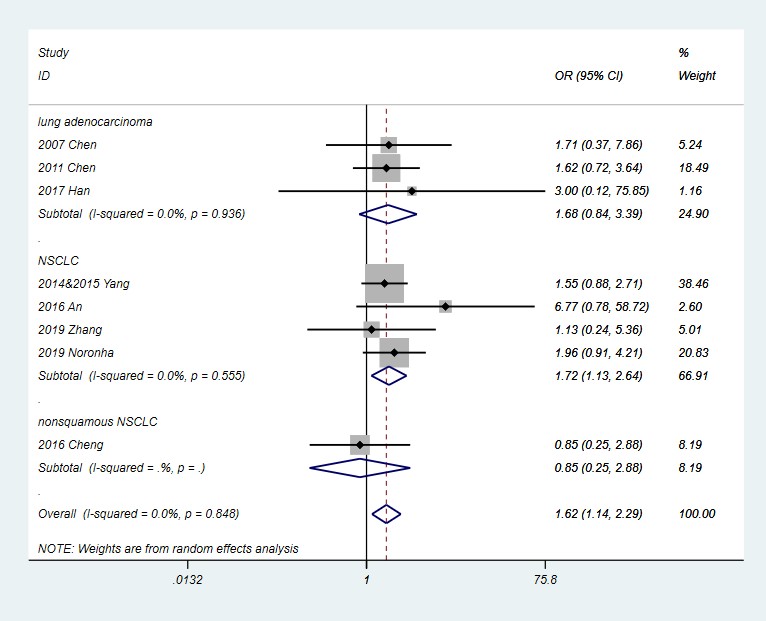


**Figure S12** Subgroup analysis for DCR (Disease Control Rate) by stage of cancer.


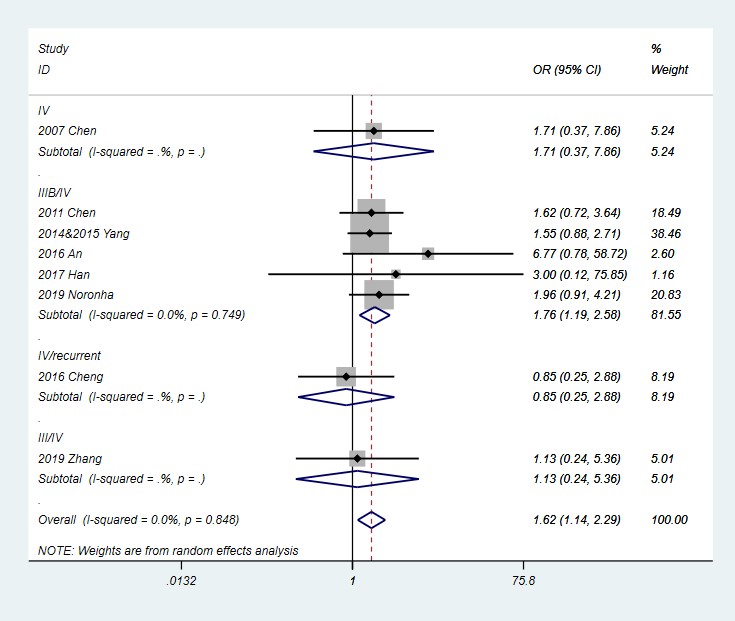


**Figure S13** Subgroup analysis for DCR (Disease Control Rate) by previous treatment.


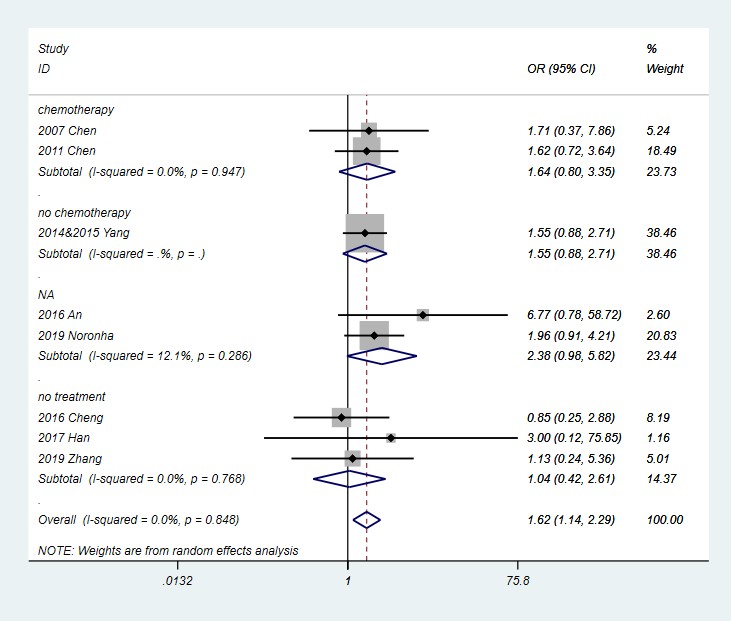


**Figure S14** Subgroup analysis for DCR (Disease Control Rate) by special type of population.


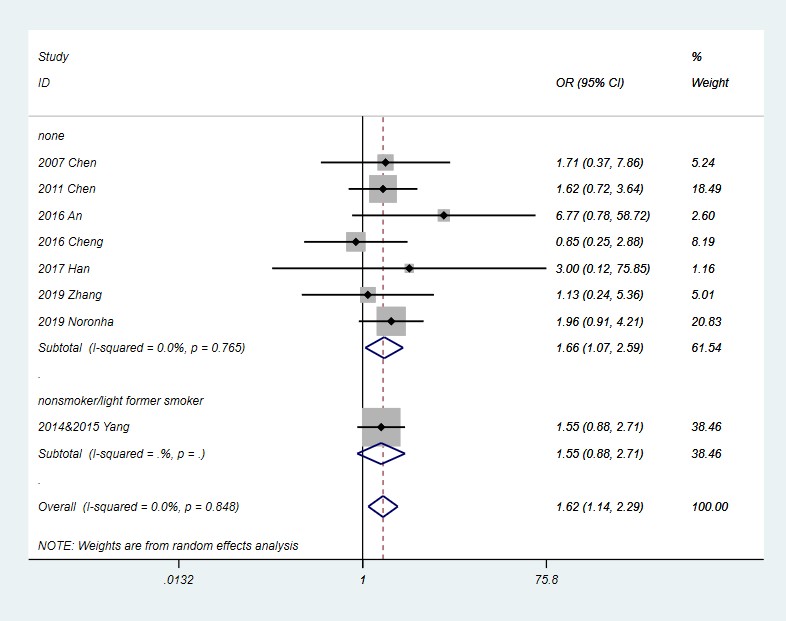


**Figure S15** Subgroup analysis for DCR (Disease Control Rate) by study design.


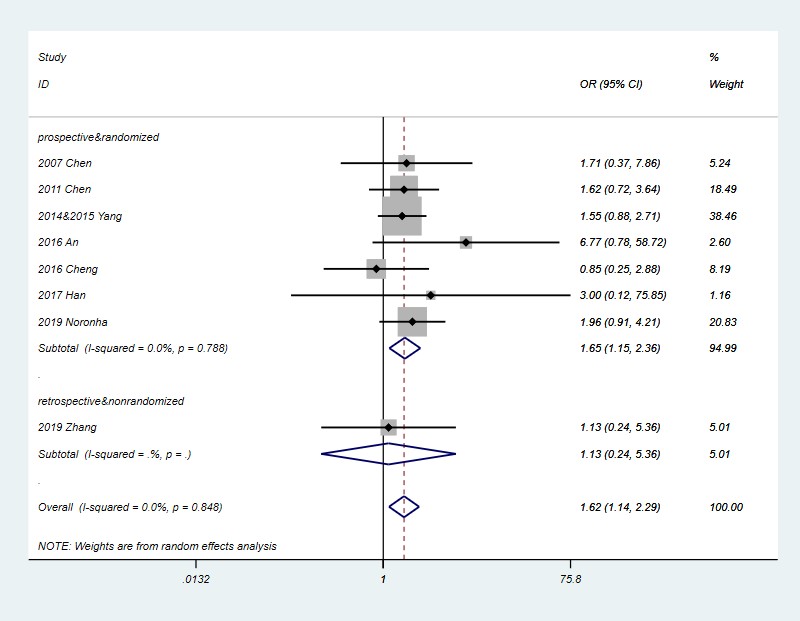


**Figure S16** Subgroup analysis for DCR (Disease Control Rate) by total number of patients.


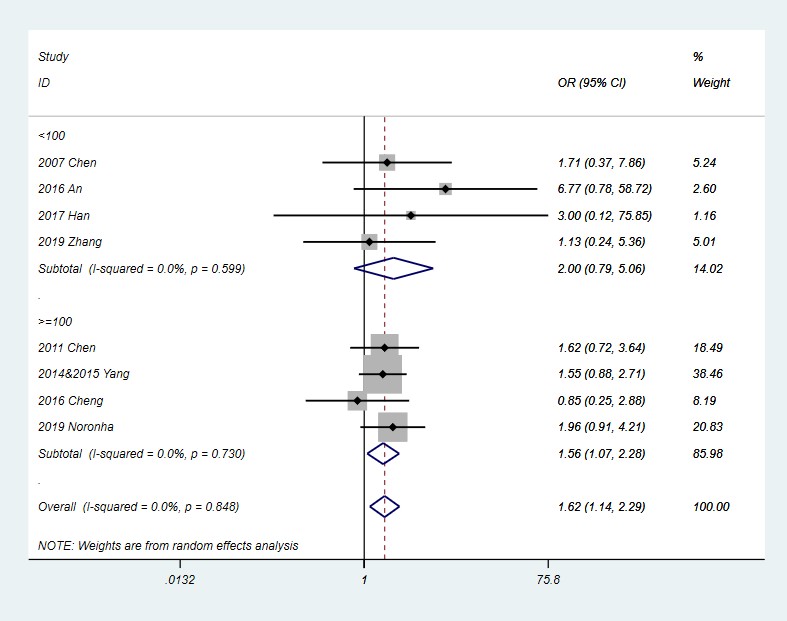


**Figure S17** Subgroup analysis for DCR (Disease Control Rate) by average age.


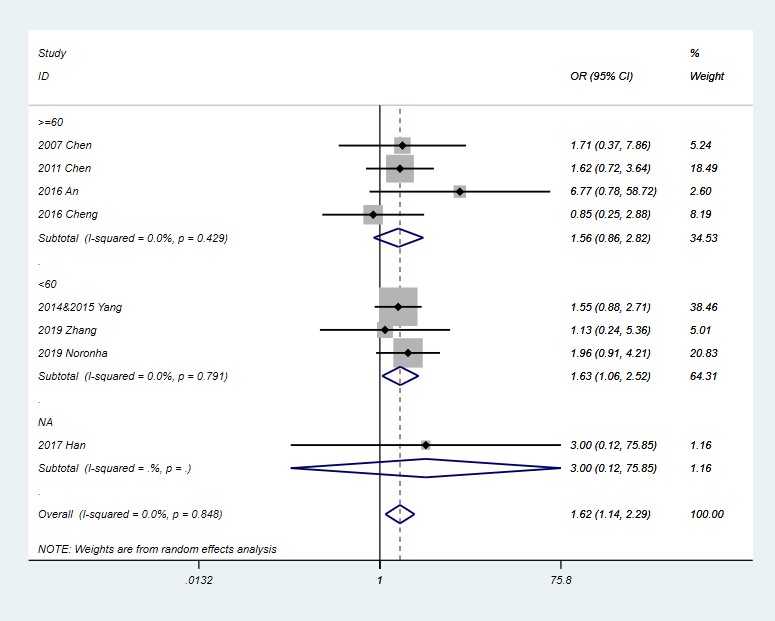


**Figure S18** Subgroup analysis for DCR (Disease Control Rate) by follow up time.


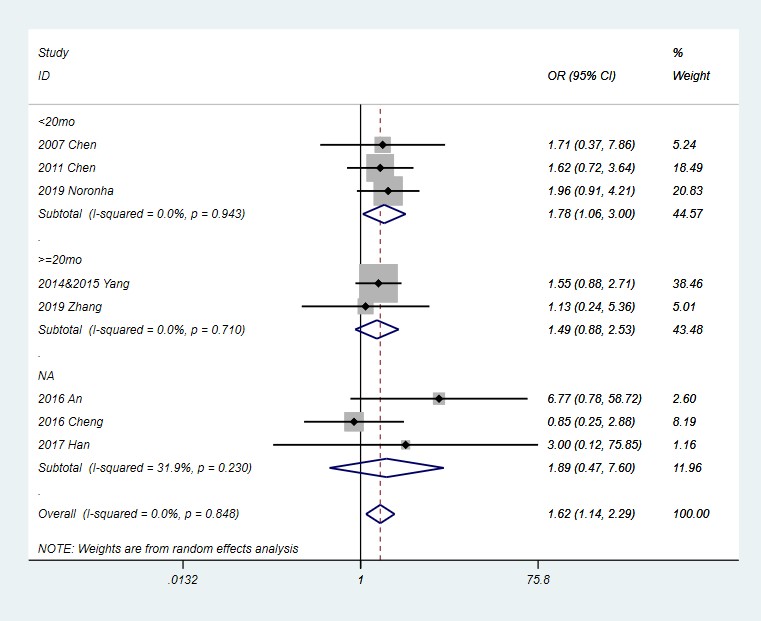


**Figure S19** Subgroup analysis for PFS (Progression-Free Survival) by study location.


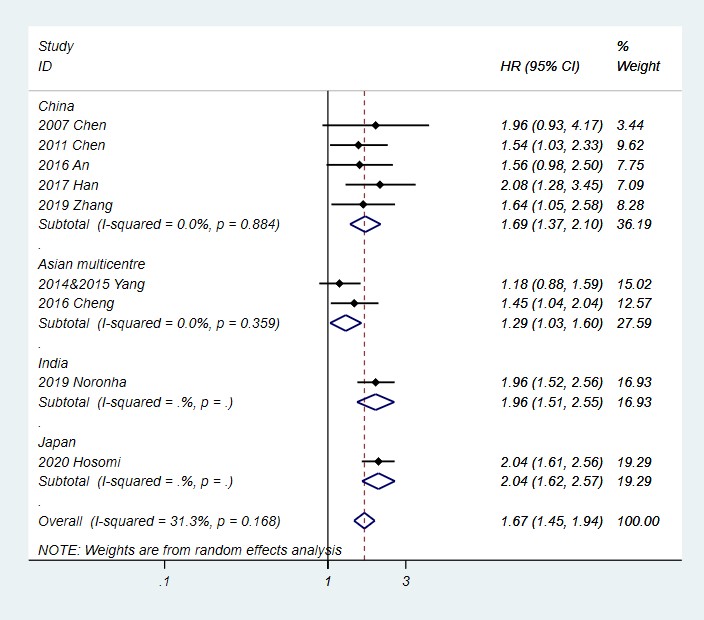


**Figure S20** Subgroup analysis for PFS (Progression-Free Survival) by type of tumor.


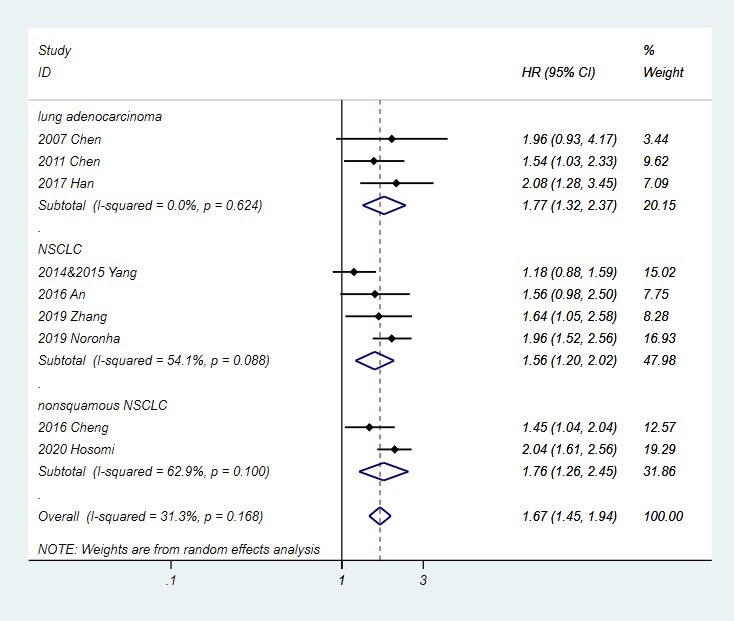


**Figure S21** Subgroup analysis for PFS (Progression-Free Survival) by stage of cancer.


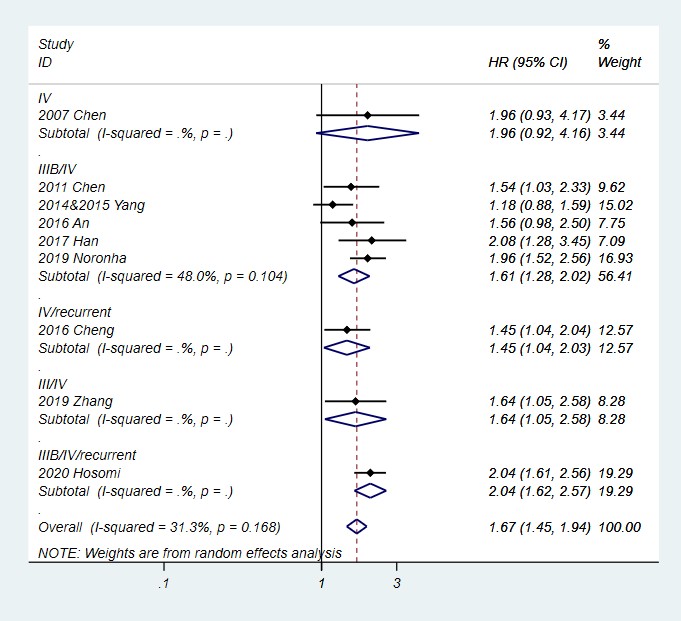


**Figure S22** Subgroup analysis for PFS (Progression-Free Survival) by previous treatment.


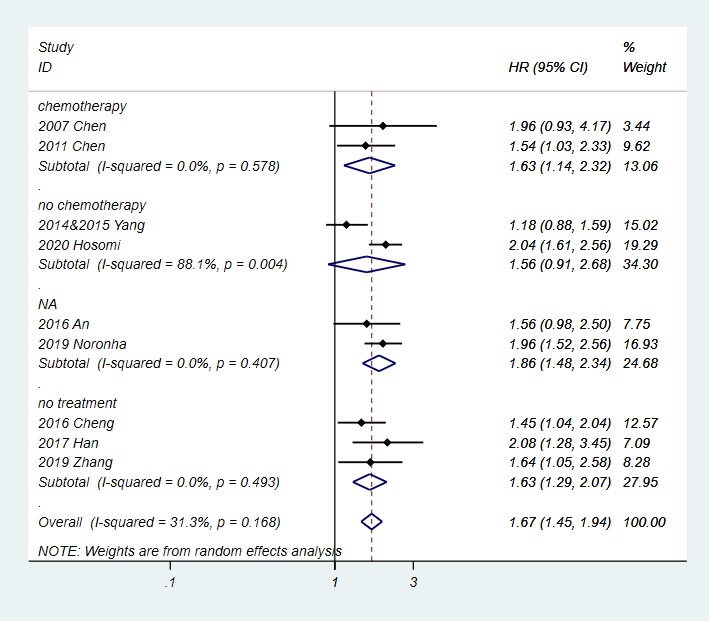


**Figure S23** Subgroup analysis for PFS (Progression-Free Survival) by special type of population.


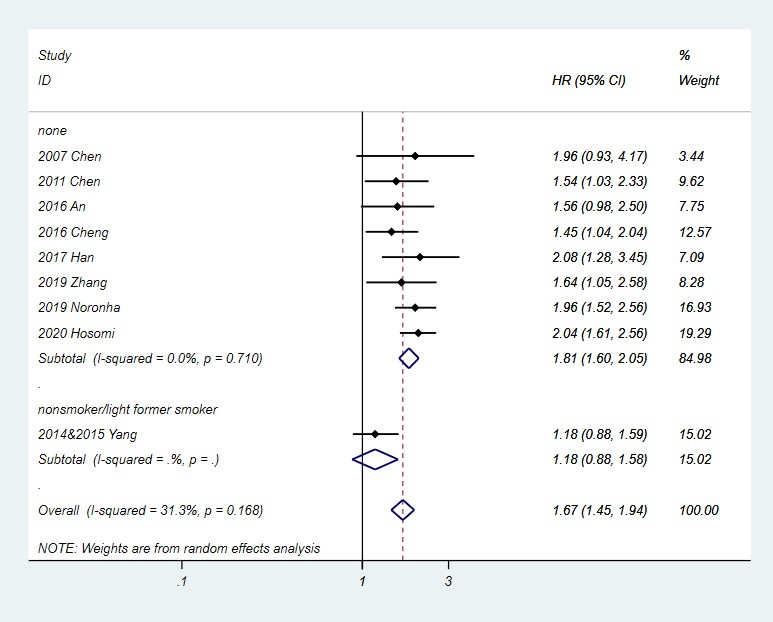


**Figure S24** Subgroup analysis for PFS (Progression-Free Survival) by study design.


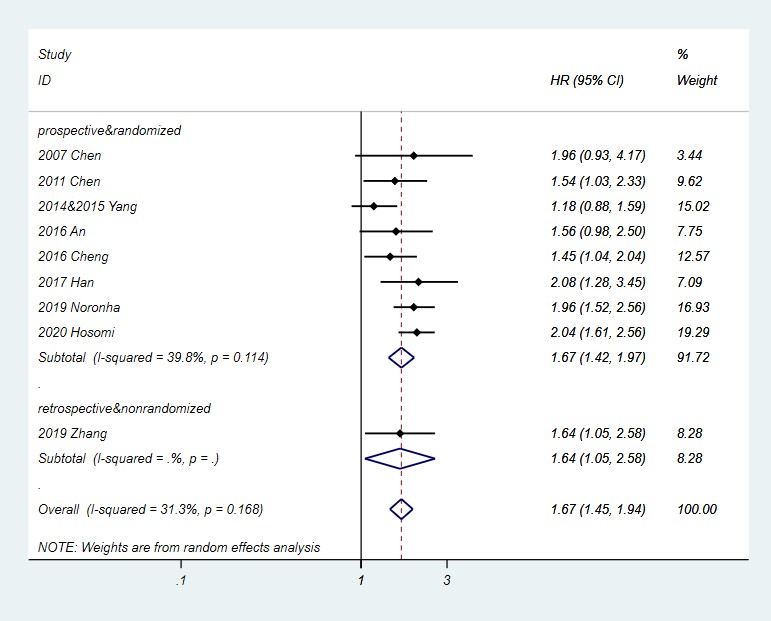


**Figure S25** Subgroup analysis for PFS (Progression-Free Survival) by total number of patients.


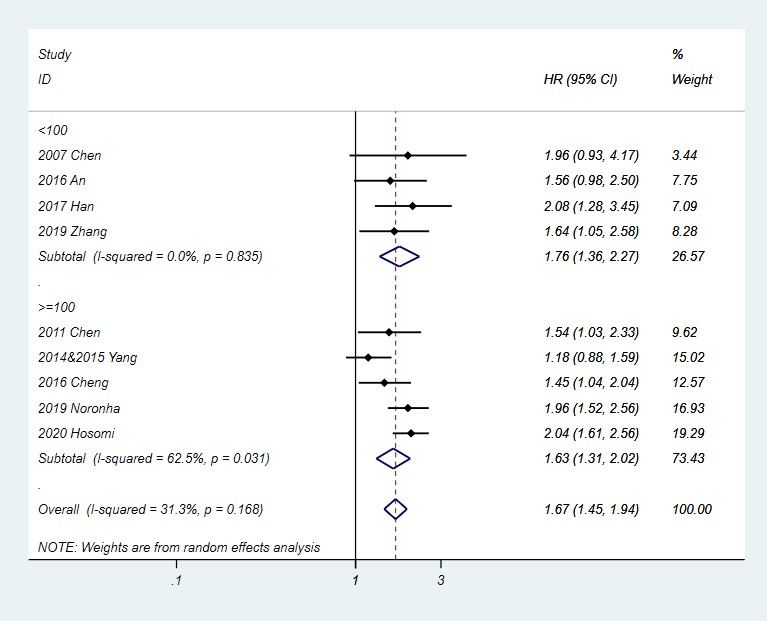


**Figure S26** Subgroup analysis for PFS (Progression-Free Survival) by average age.


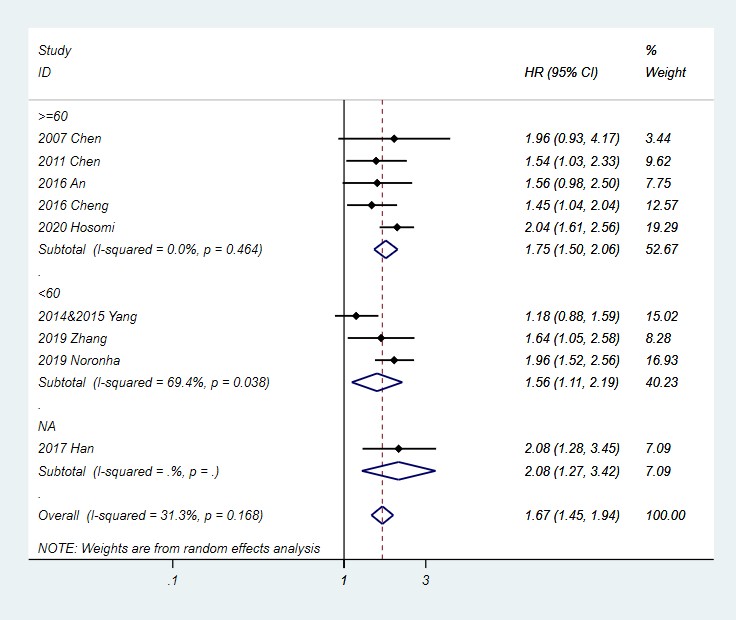


**Figure S27** Subgroup analysis for PFS (Progression-Free Survival) by follow up time.


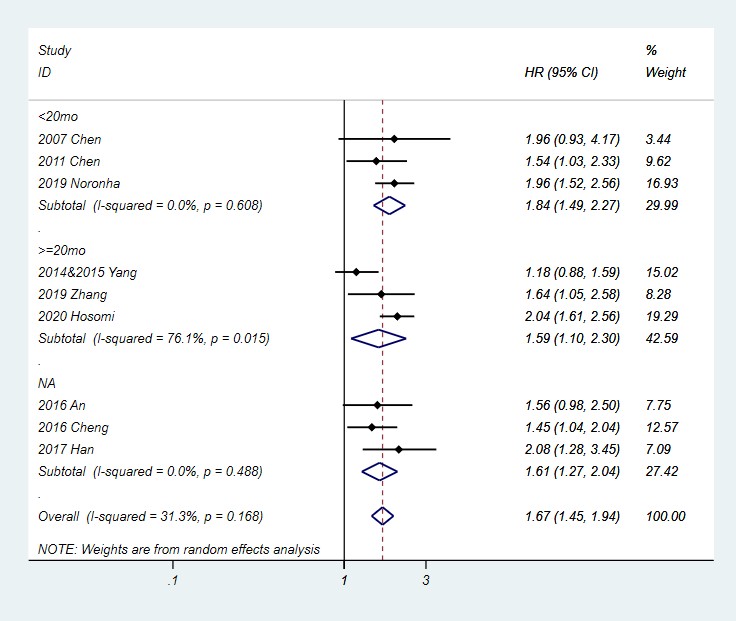


**Figure S28** Subgroup analysis for PFS (Progression-Free Survival) by whether PFS was extracted from KM curve.


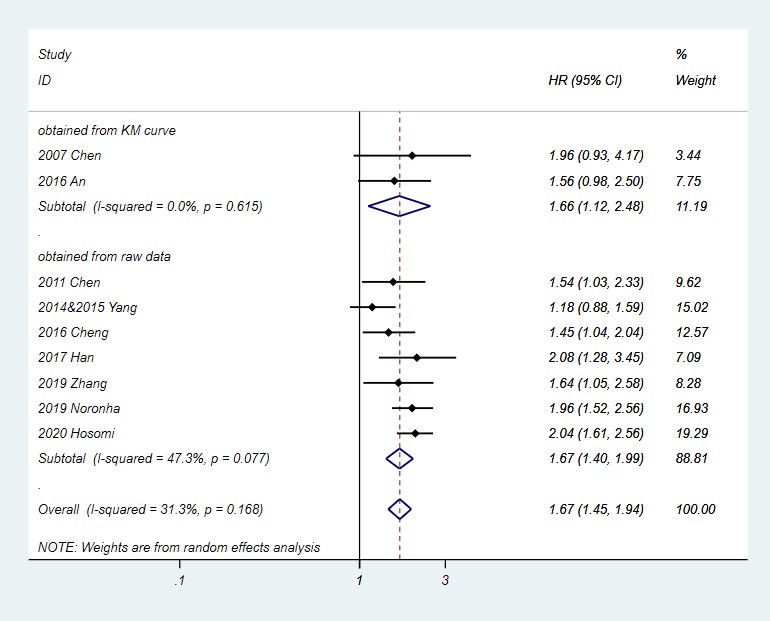


**Figure S29** Subgroup analysis for OS (Overall Survival) by study location.


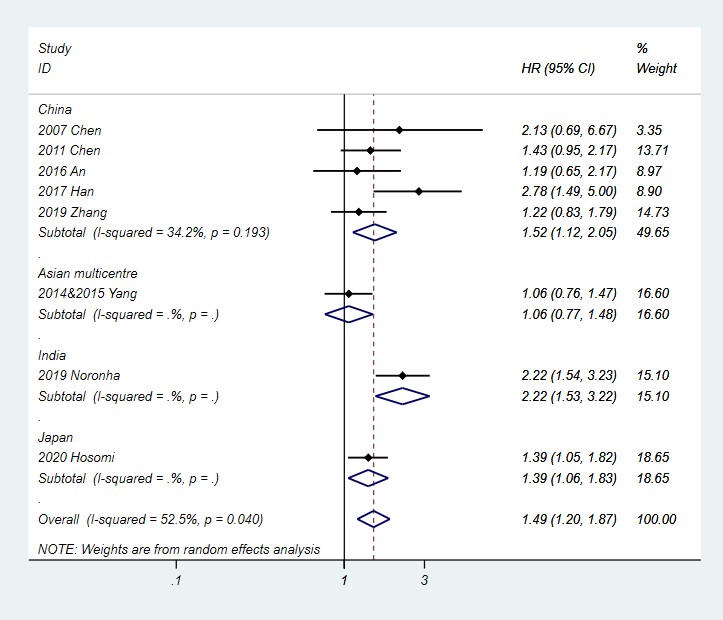


**Figure S30** Subgroup analysis for OS (Overall Survival) by type of tumor.


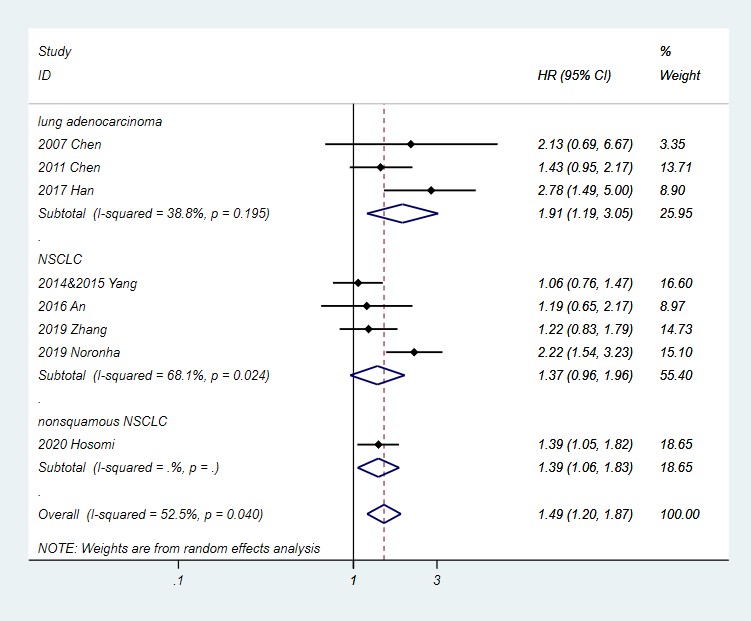


**Figure S31** Subgroup analysis for OS (Overall Survival) by stage of cancer.


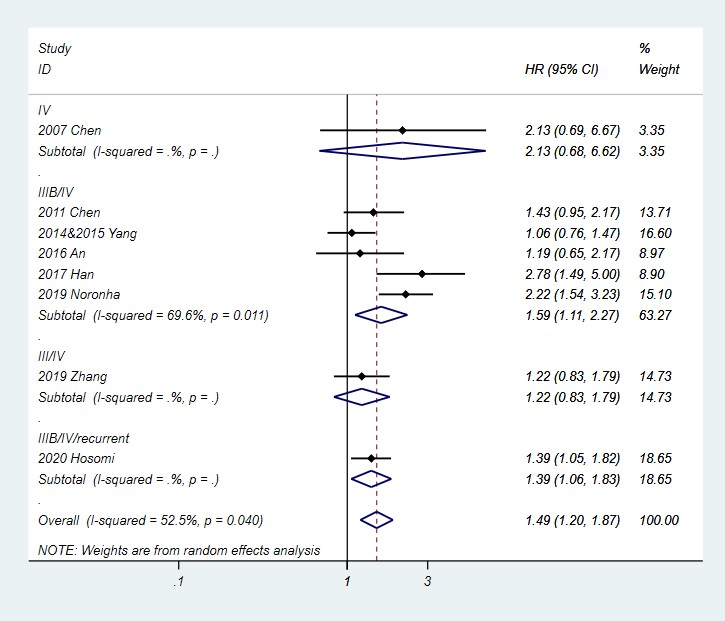


**Figure S32** Subgroup analysis for OS (Overall Survival) by previous treatment.


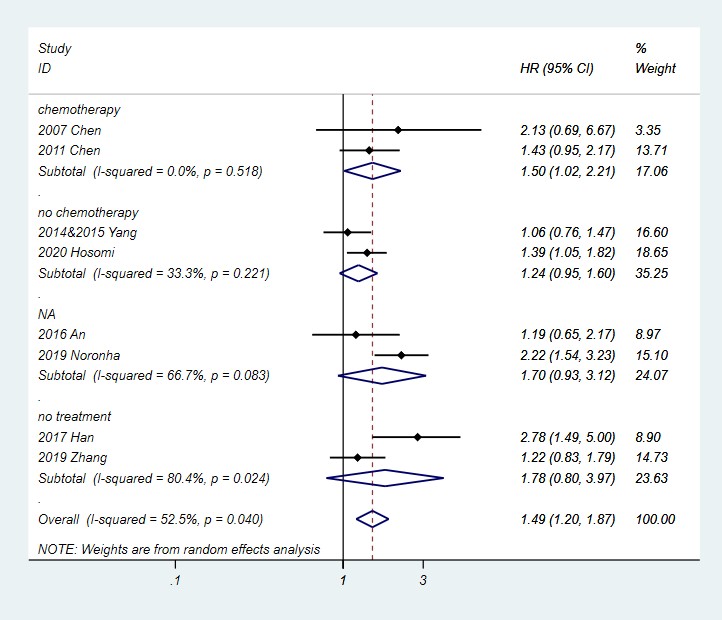


**Figure S33** Subgroup analysis for OS (Overall Survival) by special type of population.


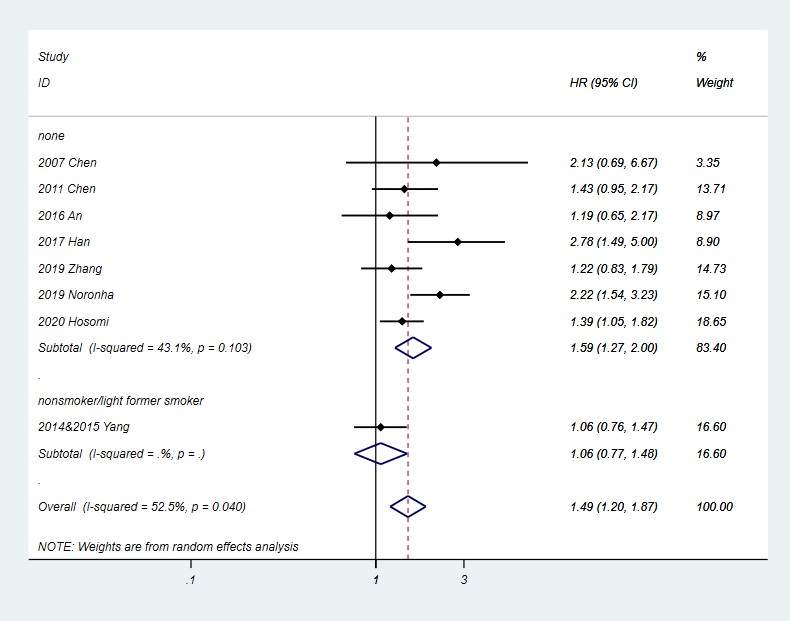


**Figure S34** Subgroup analysis for OS (Overall Survival) by study design.


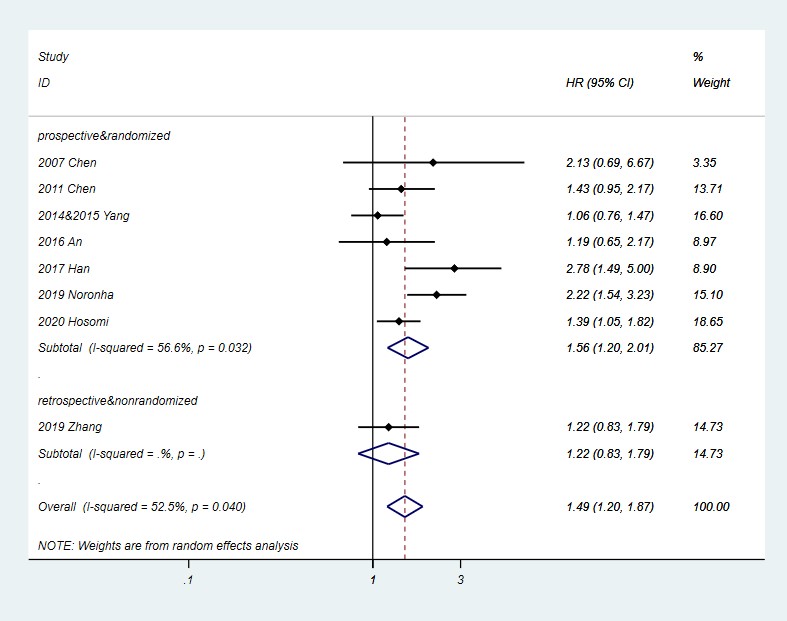


**Figure S35** Subgroup analysis for OS (Overall Survival) by total number of patients.


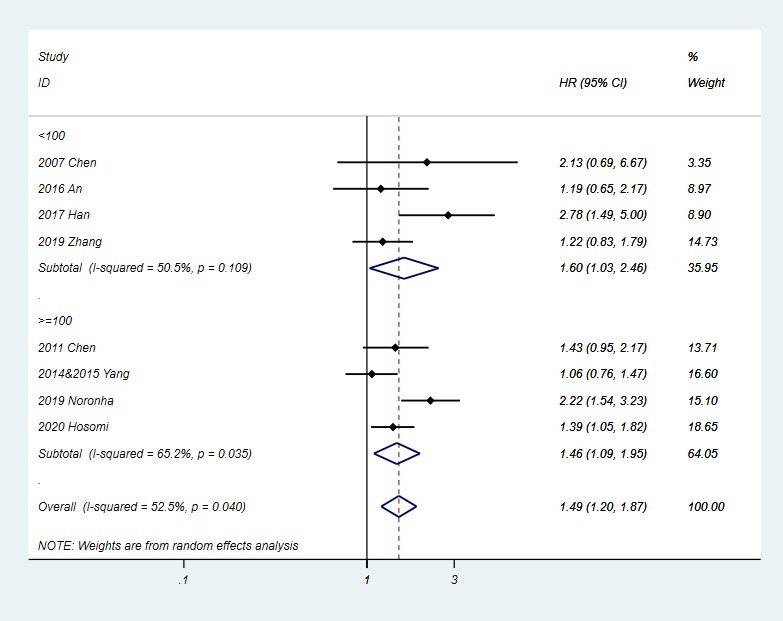


**Figure S36** Subgroup analysis for OS (Overall Survival) by average age.


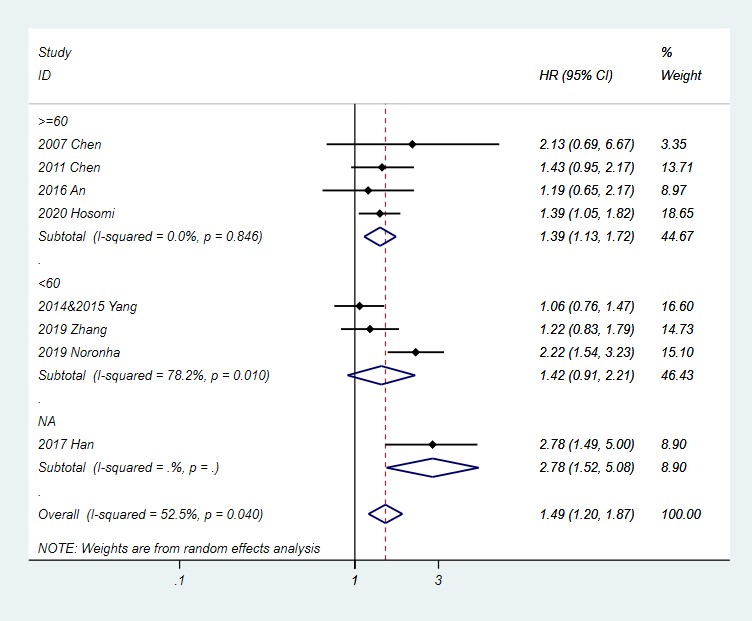


**Figure S37** Subgroup analysis for OS (Overall Survival) by follow up time.


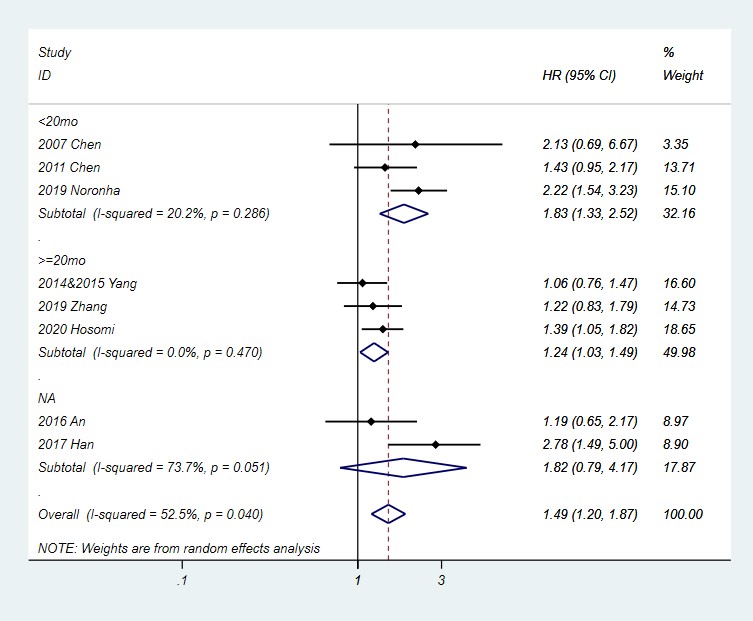


**Figure S38** Subgroup analysis for OS (Overall Srvival) by whether OS was extracted from KM curve.


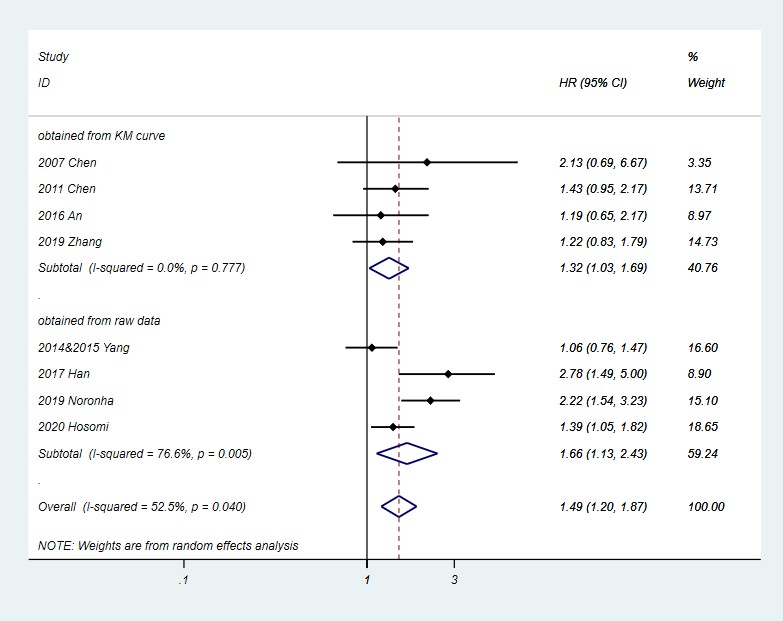

Supplement: Supplementary file 1 [file mmc1.docx]
